# Effects of proactive vs fixed community health care delivery on child health and access to care: a cluster randomised trial secondary endpoint analysis

**DOI:** 10.7189/jogh.13.04047

**Published:** 2023-04-21

**Authors:** Caroline Whidden, Kassoum Kayentao, Naimatou Koné, Jenny Liu, Mohamed Bana Traoré, Djoumé Diakité, Mama Coumaré, Mohamed Berthé, Mahamadou Guindo, Brian Greenwood, Daniel Chandramohan, Clémence Leyrat, Emily Treleaven, Ari Johnson

**Affiliations:** 1 Department of Disease Control, London School of Hygiene and Tropical Medicine, London, UK; 2 Department of Research, Monitoring & Evaluation, Muso, Bamako, Mali; 3 Malaria Research & Training Centre, Université des Sciences, des Techniques et des Technologies de Bamako, Bamako, Mali; 4 Institute for Health & Aging, University of California, San Francisco, San Francisco, California, USA; 5 Muso, Bamako, Mali; 6 Ministère de la Santé et du Développement Social, Mali; 7 Department of Medical Statistics, London School of Hygiene and Tropical Medicine, London, UK; 8 Institute for Social Research, University of Michigan, Ann Arbor, Michigan, USA; 9 Institute for Global Health Sciences, University of California, San Francisco, San Francisco, California, USA

## Abstract

**Background:**

Professional community health workers (CHWs) can help achieve universal health coverage, although evidence gaps remain on how to optimise CHW service delivery. We conducted an unblinded, parallel, cluster randomised trial in rural Mali to determine whether proactive CHW delivery reduced mortality and improved access to health care among children under five years, compared to passive delivery. Here we report the secondary access endpoints.

**Methods:**

Beginning from 26-28 February 2017, 137 village-clusters were offered care by CHWs embedded in communities who were trained, paid, supervised, and integrated into a reinforced public-sector health system that did not charge user fees. Clusters were randomised (stratified on primary health centre catchment and distance) to care during CHWs during door-to-door home visits (intervention) or based at a fixed village site (control). We measured outcomes at baseline, 12-, 24-, and 36-month time points with surveys administered to all resident women aged 15-49 years. We used logistic regression with cluster-level random effects to estimate intention-to-treat and per-protocol effects over time on prompt (24-hour) treatment within the health sector.

**Results:**

Follow-up surveys between February 2018 and April 2020 generated 20 105 child-year observations. Across arms, prompt health sector treatment more than doubled compared to baseline. At 12 months, children in intervention clusters had 22% higher odds of receiving prompt health sector treatment than those in control (cluster-specific adjusted odds ratio (aOR) = 1.22; 95% confidence interval (CI) = 1.06, 1.41, *P* = 0.005), or 4.7 percentage points higher (adjusted risk difference (aRD) = 0.047; 95% CI = 0.014, 0.080). We found no evidence of an effect at 24 or 36 months.

**Conclusions:**

CHW-led health system redesign likely drove the 2-fold increase in rapid child access to care. In this context, proactive home visits further improved early access during the first year but waned afterwards.

**Registration:**

ClinicalTrials.gov NCT02694055.

Ensuring that all people have access to quality health services without financial hardship is central to achieving universal health coverage (UHC) and other health-related targets of the Sustainable Development Goal (SDGs). Despite progress to date, up to one-third of the world’s population may not benefit from UHC by 2030 [1]. Achieving these goals requires a fundamental shift in how primary care is organised, managed, and delivered.

Community health workers (CHWs) have the potential to contribute to the diverse, sustainable health workforce required to deliver integrated, people-centred primary care [1]. Low and middle-income countries (LMICs) are increasingly adopting integrated community case management (iCCM) (comprising the diagnosis, treatment, and referral in the community for childhood malaria, diarrhoea, pneumonia, acute malnutrition, and/or newborn illnesses [2]) as a CHW-led strategy to improve service coverage and health outcomes among children under five years of age [3,4]. This scale-up is motivated by substantial evidence that CHWs can deliver a range of preventive and curative primary care services [5-7], including community case management for malaria [8,9], diarrhoea [10], and pneumonia [10-12] to increase utilisation, improve health, and reduce mortality among under-five children in many settings.

However, iCCM programme design and implementation vary greatly between settings, to variable effects [13,14]. Evaluations of scaled iCCM in Burkina Faso, Ethiopia, and Malawi found implementation shortcomings related to CHW training and deployment, health systems, and community mobilisation, and no effects on care-seeking, treatment coverage, or child mortality [15-17]. A systematic review of iCCM found moderate quality evidence that care-seeking from an appropriate provider increased by 68%, compared to facility-based care, yet inconsistent effects on the receipt of adequate treatment from an appropriate provider and under-five mortality among included studies, few of which included payment, supervision, or information systems to support CHWs [18].

Optimising iCCM means moving beyond training and deploying CHWs to ensure that these frontline health workers are integrated into and adequately supported by the health system [18]. The World Health Organization (WHO) guidelines released in 2018 recommend CHW remuneration, functioning referral systems, supply chain management, and supportive supervision, among other health system enablers [19]. However, existing gaps in the evidence do not allow for the recommendation of specific programme design features such as CHW workflow or approaches by which community-based services like iCCM are delivered [18,19].

Across sub-Saharan Africa, including in Mali, CHWs are stationed in community health sites to provide iCCM and other community-based services to patients who seek care. An alternative to this conventional, passive approach to service delivery is a proactive workflow in which CHWs conduct routine door-to-door home visits, searching for and identifying prospective patients. Proactively offering promotive, preventive, and curative services at patients’ doorsteps may improve community engagement, service coverage, and treatment outcomes, and especially the speed with which evaluation and treatment are received.

Ensuring prompt treatment, particularly within the crucial 24-hour window after symptom onset in children under five, is a cornerstone of global iCCM and malaria control programmes. A meta-analysis estimated that almost half of severe childhood malarial anaemia cases in the included studies could have been averted if children had accessed facility-based treatment within the first day of symptom onset [20]. From Brazil to Uganda, studies using verbal and social autopsy data have uncovered how delays at various points along the trajectory to care contribute to child death due to diarrhoea, acute respiratory infection, and newborn illnesses [21-23].

Based on existing evidence, it is uncertain whether proactive case-finding home visits by CHWs can improve prompt treatment and reduce the prevalence of infectious diseases or under-five mortality [24]. We implemented a cluster randomised trial to evaluate the effects of proactive CHW home visits on child mortality (primary trial endpoint) and access to care in rural, central Mali [25]. The primary trial endpoint results will be reported separately (unpublished data). Here we report the secondary trial endpoint analysis on child health and service utilisation over the three-year trial period, including the receipt of prompt treatment within the health sector, receipt of recommended case management according to iCCM protocols, and the prevalence of common childhood illnesses in this context. We assessed whether effects differed according to population size, distance to primary health centre (PHC), or household wealth, to determine the equity of this approach.

## METHODS

### Study design and participants

We conducted a pragmatic, cluster randomised controlled trial, with a stratified, two-arm, parallel group design in a rural setting in the Bankass health district of central Mali’s Mopti region. The district, served by one public secondary referral hospital and 22 PHCs was chosen in partnership with the Malian Ministry of Health and Social Development based on its high under-five mortality and low health care utilisation [26,27], with few concurrent health interventions and a high interest from local authorities in collaborating. From initial geo-mapping across seven contiguous PHC catchment areas, villages and hamlets one kilometre or less apart were grouped into clusters. We randomised clusters in a 1:1 allocation to intervention and control arms to receive CHW services delivered via proactive home visits (n = 69 clusters) or only at a fixed community health site (n = 68 clusters), respectively.

To assess outcomes, we censused all permanent residents and surveyed all resident women aged 15 to 49 years at baseline and annually at 12, 24, and 36 months. Respondents provided written, informed consent (or assent, if aged 15 to 17 years and unmarried) at their first enrolment and were included in follow-up surveys if present (including those who were aged above 49 years). Any individual who sought care from study providers was eligible to receive health care throughout the trial, regardless of residency, survey enrolment, or arm assignment.

### Randomisation and masking

We used the timeline cluster graphical tool to describe the sequencing and blinding of the different recruitment, randomisation, and assessment procedures implemented during the trial, and whether they were conducted at the cluster or participant level, or both (Figure S1 in the **Online Supplementary Document**) [28]. We stratified the randomization by health catchment area and distance to the nearest PHC. In total, we had 21 strata. Each of the seven catchment areas had three strata: one for the cluster where the PHC was located, one for clusters within five kilometres from the PHC, and one for clusters beyond this distance. Given the nature of the intervention, we could not blind the participants, providers, or outcome assessors. Statisticians were blinded throughout the trial, until the data were fully cleaned and locked by the Data Safety & Monitoring Board (DSMB).

### Procedures

In each cluster, community leaders nominated individuals aged 18 to 45 years who could read and write in French to be trained, selected, and deployed as CHWs. Nominees were divided by study arm and trained separately over six weeks, with annual one-week refresher training, based on the same clinical protocols (that covered preventive and curative primary care for reproductive, maternal, newborn, and child health, including iCCM for diarrhoea, pneumonia, malaria, acute malnutrition, and newborn illnesses) and the delivery approach to which their clusters were allocated. CHWs were ultimately selected based on a post-training evaluation and deployed to serve approximately 700 people, in line with Mali’s 2016-2020 national community health strategy [29].

CHWs in the intervention arm were instructed to conduct door-to-door proactive case-finding home visits for at least two hours per day, six days per week, with the goal of visiting every household at least twice per month. In the control arm, CHWs were instructed to station themselves at community health sites for four hours per day, six days per week, to provide the same package of services to care-seeking patients. CHWs in both arms were expected to be available on-call to provide care as needed, at all times.

CHWs in both arms received the same systems support, in accordance with WHO guidelines [19]. All CHWs signed contracts with the Community Health Associations (ASACO) that manage public-sector PHCs, received part-time salaries and benefits that met local minimum wage requirements, and had performance-based opportunities to advance into the cadre of dedicated CHW supervisors. All CHWs received individual, monthly supervision that included house calls without the CHW to solicit patients’ perspectives, direct observation while conducting home visits or stationed at their site (depending on which arm they were allocated to), and one-on-one feedback aided by a personalised performance dashboard [30]. Dedicated supervisors also held group supervision meetings twice per month, separately by arm. Supervisors monitored CHWs’ supplies and equipment, including the CHW smartphone-based mobile application for recording patient encounters. All CHWs were supported by a functioning referral system, as all study PHCs received reinforcements in infrastructure (e.g. waiting area, separate general and maternity wards), equipment, supplies, and human resources (e.g. recruitments and training). Finally, user fees were removed at all points of care, from CHW to tertiary hospital, for patients in both arms. The redesigned CHW-led health system in both arms was launched February 26-28, 2017.

We assessed the outcomes at baseline (December 2016 to January 2017) and approximately 12 (February to March 2018), 24 (March to May 2019), and 36 months (January to April 2020) via surveys administered at respondents’ homes by female surveyors who were neither community residents nor involved in health care delivery. We adapted the household and women’s surveys from Mali’s Demographic and Health Survey (DHS) and programmed in Open Data Kit. They included a household roster (census) and modules on migration, mortality, and socio-economic characteristics. The women’s survey included socio-demographic characteristics, current contraceptive use, most recent pregnancy and childbirth, lifetime birth history, and symptoms and service utilisation in the two weeks preceding the survey for all the woman’s co-residing children under five years of age.

### Outcomes

We assessed all outcomes using the women’s survey, measured at the child level and analysed at the child-year level. The primary outcome was prompt treatment within the health sector, defined as a child aged 0-59 months with any symptom at any time in the two weeks preceding the survey who had received CHW or public or private health centre evaluation and any treatment, including traditional or home remedies, the same or next day after symptom onset. Secondary outcomes included any prompt treatment (from any source), health sector evaluation (CHW or public or private health centre consultation, with or without prompt treatment), and any care (inside or outside the home). As the intervention was designed to improve UHC, we defined (in an appendix to the trial statistical analysis plan that was approved by the DSMB prior to unblinding) composite utilisation outcomes that assessed access to care for all sick children, regardless of illness. Consistent with endpoints defined in the trial protocol [25], we included as secondary outcomes recommended case management and prompt. According to iCCM clinical protocols [2], we defined recommended case management as a child aged 3-59 months with fever, and/or diarrhoea without blood, and/or cough with fast breathing (i.e., suspected pneumonia) who had received a rapid diagnostic test for malaria, and/or oral rehydration solution (ORS) and zinc, and/or antibiotics, respectively; newborns were excluded as their clinical protocol was different. We were unable, however, to conduct stratified analyses by illness due to fewer clusters with cases and events per illness. To contextualise the access to care results and assess intervention effects on child morbidity, we also included the prevalence of fever, diarrhoea, cough, and suspected pneumonia in the two weeks preceding the survey among all children under five years.

### Statistical analysis

We based the sample size calculation, planned interim analyses, and stopping guidelines on the trial’s primary endpoint (deaths among children under five years of age per 1000 person-years at risk of mortality), as reported in the protocol [25].

For all ten outcomes, we first generated cluster-specific summaries (means) by calculating the proportion in each cluster at each time point and plotting the median per arm and cluster-level variability over time. We then estimated the intervention effects using the following mixed effects logistic regression model on the intention-to-treat (ITT) population:



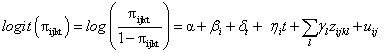



Here, π_ijkt_ is the probability for the *k^th^* individual in the *j^th^* cluster in the *i^th^* treatment arm, at the *t^th^* time point. *α* is the constant, representing the mean outcome among individuals in the control arm. (*β_i_*) is the cluster-specific odds ratio (OR_CS_) representing the outcome in the intervention arm (*i =* 1) compared to the control arm (*i =* 0). *δ_t_* represents the time effect, with *t =* 1, 2, 3 corresponding to three consecutive follow-up surveys. *η_i_t* is the interaction term that estimates the differential effect of the intervention arm relative to the control arm across the three time points. For each outcome, we fit an additional model without the interaction term that estimated an overall cluster-specific effect throughout the three-year trial, controlling for the linear effect of time. *γ_l_* is a vector of the estimated coefficients for the following set of covariates, represented by *z*_ijkl_ (*l = 1,2,…,L*): a cluster-level summary of the baseline value of the outcome, baseline cluster-level summaries of sample characteristics that were deemed imbalanced at baseline and likely to influence the outcome, individual’s age and sex, and variables on which randomisation was stratified. Cluster-level random effects, , accounted for within-cluster correlation. For prevalence outcomes, we included an additional random intercept, *ν_ijk_*, to account for repeated measure and within-individual correlation over time. We conducted all statistical analyses using Stata version 15 (StataCorp, College Station TX, USA). We reported the results following the CONSORT guidelines [31], including the presentation of both relative and absolute effect sizes (using the margins post-estimation command) and the intracluster correlation coefficient (ICC) per arm (taking the rho coefficient of models run separately by arm, or using the estat post-estimation command with multilevel models).

We assessed heterogeneous treatment effects by fitting models that included an interaction term between an arm and prespecified effect modifiers at each time point separately (to facilitate the interpretation of interaction effects; prespecified analysis) and during the three-year period overall (controlling for the linear effect of time; post-hoc analysis). We used likelihood ratio tests to determine if there was evidence to reject the assumption of no interaction/effect modification. As potential modifiers, baseline cluster population size and distance to PHC were chosen to critically examine design features of Mali’s community health strategy [29], which recommends one CHW per 700 people only in villages more than five kilometres away from a PHC. Household wealth was chosen to permit an equity sub-analysis, examining differential effects for children living in households in the poorest wealth quintile.

We conducted a prespecified per-protocol subgroup analysis by excluding (from the main model/equation above) child-year observations in the intervention arm if no female respondent in the household reported receiving at least two CHW home visits in the month preceding the survey, and then by additionally excluding child-year observations in the control arm if any female respondent in the household reported a home visit in the last month.

The main intervention effect models used complete-case analysis. However, due to missing treatment data at the 24-month time point caused by a data capture coding error, we performed multiple imputation by chained equations (MICE) in sensitivity analyses on related outcomes: primary outcome, any prompt treatment, recommended case management, and prompt, recommended case management. Furthermore, because missing outcome data exceeded the predefined 10% threshold for the 24-month subset, we performed MICE prior to assessing heterogeneous treatment effects at 24 months. Due to correlation between outcomes, we ran separate MICE models, each generating 20 imputed data sets. We included all variables and interaction terms that appeared in one or more subsequent regression analyses and two auxiliary variables (any treatment received and CHW care received) associated with missing data. Due to strong clustering for outcomes and missing data, we were unable to impute data separately by cluster or include indicator variables for clusters. Instead, we captured between-cluster variability by including all baseline cluster-level covariates and outcome summaries when creating imputations.

### Patient and public involvement

We involved national and district level authorities from the Malian Ministry of Health and Social Development in the study design, implementation, and dissemination. We chose research questions (including an embedded costing analysis) and outcomes for the trial (including the primary outcome on under-five mortality) that were of key interest to our government partners. We also involved national and district health authorities in study site selection, including both the rural district within the country and the seven PHC catchment areas within the district. Within each catchment area, we held public consultation meetings with community representatives, including village chiefs and their advisors, women’s and youth association leaders, religious leaders, and politico-administrative authorities (such as mayors, PHC directors, and ASACOs), where we discussed and obtained verbal permission to conduct the trial. Communities nominated CHW candidates who participated in the training and provided a fixed health site for control arm CHWs, as well as a house if the CHW was not a resident of the village-cluster.

Once we conducted the analysis on the trial’s primary and secondary endpoints, including child health and access to care, we held results dissemination workshops with local, district, regional, and national level stakeholders, starting at the district and local level with community representatives (as listed above), including study CHWs and their dedicated supervisors.

### Role of the funding source

The funders had no role in study design, data collection, analysis, interpretation, or writing of this paper. The corresponding author had full access to all the data in the study and had final responsibility for the decision to submit for publication.

### Ethics

The trial received ethical approval from the Faculty of Medicine, Pharmacy and Odonto-Stomatology Ethics Committee at the Université des Sciences, des Techniques et des Technologies of Bamako (Ref: 2016/03/CE/FMPOS). Secondary analysis of trial data was approved by the Observational/Interventions Research Ethics Committee at the London School of Hygiene & Tropical Medicine (Ref: 13832) and exempted by the University of California, San Francisco (Ref: 154824)

## RESULTS

Baseline data collection covered 137 clusters, censused 99 576 people, and surveyed 15 884 women of reproductive age who provided outcome data on 15 855 children under five years (Figure S2 in the **Online Supplementary Document**). Clusters , children under five years of age, sick children under five years, and children aged 3-59 months with iCCM illnesses had similar characteristics between arms at baseline (**Table 1**, Table S1-S3 in the **Online Supplementary Document**). All clusters contributed observations to the analysis. However, between the 12- and 24-month surveys, due to escalating violent conflict in the study area, three intervention and three control clusters, all relatively small and remote , were lost to follow-up (Table S4 and Figure S2 in the **Online Supplementary Document**). Sample characteristics were similar between observations with complete vs missing outcome data (Tables S5-S7 in the **Online Supplementary Document**). Analyses included 46 789 child-year observations, 20 105 sick child-year observations, and 15 278 child-year observations with iCCM illnesses during the three-year trial period. Among all child-year observations, 57% were repeated measures on the same child; 28% of sick child-year and 22% of child-year observations with iCCM illnesses were repeated measures.

**Table 1:** Baseline cluster-level characteristics and summaries of the outcomes of interest*

	Intervention	Control
**Characteristics, n (%)**	n = 69 clusters	n = 68 clusters
Population size, median (IQR)	532 (305.0-1087.0)	564 (243.5-984.0)
<700	38 (55.1)	40 (58.8)
≥700	31 (44.9)	28 (41.2)
Distance from PHC in kilometres, median (IQR)	6.3 (4.2-8.6)	5.8 (3.5-8.6)
≤5.0	28 (40.6)	29 (42.7)
>5.0	41 (59.4)	39 (57.4)
Topography		
None	63 (91.3)	64 (94.1)
On clifftop	1 (1.5)	2 (2.9)
PHC inaccessible during rainy season (June, July, August)	5 (7.3)	2 (2.9)
CHW services available†		
None	51 (73.9)	51 (75.)
Satellite village	14 (20.3)	14 (20.6)
Posted village	4 (5.8)	3 (4.4)
PHC catchment area		
Dimbal	15 (21.7)	15 (22.1)
Lessagou	14 (20.3)	12 (17.7)
Doundé	8 (11.6)	7 (10.3)
Ende	2 (2.9)	3 (4.4)
Soubala	11 (15.9)	13 (19.1)
Kanibozon	9 (13.0)	8 (11.8)
Koulongon	10 (14.5)	10 (14.7)
**Outcomes, median (IQR)**		
Prevalence	n = 69 clusters	n = 68 clusters
Fever	0.12 (0.05-0.24)	0.12 (0.06-0.22)
Diarrhoea	0.14 (0.08-0.28)	0.16 (0.08-0.26)
Cough	0.10 (0.06-0.15)	0.11 (0.04-0.18)
Suspected pneumonia	0.03 (0-0.05)	0.03 (0.00-0.05)
Health care utilisation, median (IQR)	n = 67 clusters‡	n = 68 clusters
Prompt treatment within health sector	0.19 (0.09-0.31)	0.19 (0.06-0.27)
Any prompt treatment	0.48 (0.29-0.60)	0.45 (0.33-0.55)
Health sector evaluation	0.21 (0.14-0.37)	0.20 (0.08-0.33)
Any care	0.55 (0.38-0.68)	0.50 (0.42-0.66)
Recommended case management, median (IQR)	n = 67 clusters	n = 68 clusters
Recommended case management	0.21 (0.93-0.30)	0.16 (0.08-0.27)
Prompt, recommended case management	0.16 (0.08-0.25)	0.12 (0.05-0.21)

CHW – community health care workers, IQR – interquartile range, PHC – primary health centre

*Data are presented as n (%) unless otherwise specified.

†CHWs are stationed/posted in some communities at baseline and may also serve members from neighbouring/satellite communities.

‡There are two intervention clusters with no sick children at baseline.

**Figure 1:**
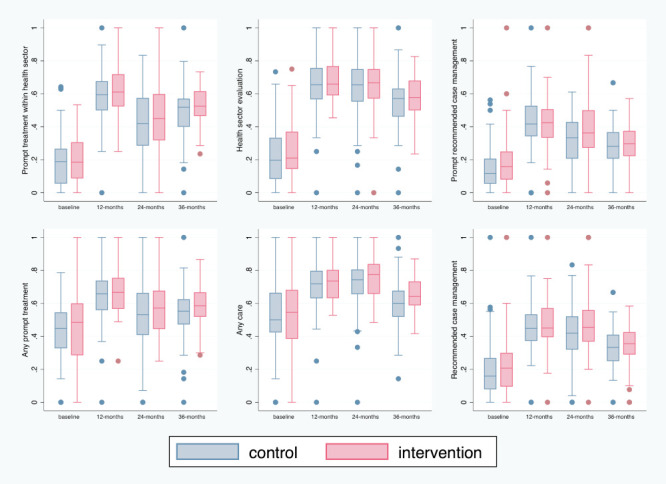
Box plots representing the variability in cluster summaries of the primary and secondary health care utilisation outcomes in intervention and control arms at each time point.

Prompt treatment within the health sector increased from a median of 19% across all clusters at baseline to 61% at 12 months, 44% at 24 months, and 52% at 36 months, with similar trends in both arms (**Figure 1**). Similarly, one in five children at baseline received health sector evaluation, which increased to two-thirds at 12 and 24 months and over one-half at 36 months across arms. Recommended case management also increased two-fold compared to baseline, and similarly between arms (**Figure 1**), but did not reach half of the children with iCCM illnesses during the trial. Whether sick children received any prompt treatment (from any source) or any care varied considerably between clusters at baseline, but less so during the trial, reaching as many as 66% or 72%, respectively, at 12 months across arms.

At the 12-month follow-up, the odds of receiving prompt treatment within the health sector were 22% higher in intervention compared to control clusters (AOR_CS_ = 1.22; 95% confidence interval (CI) = 1.06, 1.41, *P* = 0.005) (**Table 2**). At 12 months, children in intervention clusters were 4.7 percentage points more likely to receive prompt health sector treatment than those in control clusters (adjusted risk difference (ARD) = 0.047; 95% CI = 0.014, 0.080). However, there was no evidence of an intervention effect at 24 or 36 months. Findings were similar for any prompt treatment. Furthermore, the results were consistent in sensitivity analyses dealing with missing data, including multiple imputation (Table S8 in the **Online Supplementary Document**). The ICC for the primary outcome was 0.017 (95% CIs = 0.010, 0.029) in the intervention arm and 0.019 (95% CI = 0.010, 0.035) in the control arm.

**Table 2:** Cluster-specific intervention effects on primary and secondary health care utilisation outcomes, including absolute risks in each arm, during the three-year trial period overall and at each follow-up time point*

	ARC	ARI	C vs I, AOR_CS_ (95% CI)	*P*-value
**Prompt treatment within the health sector (n = 18 765)**				
Overall†	0.52	0.55	1.10 (0.98-1.24)	0.103
Time point‡				
12 mo	0.58	0.62	1.22 (1.06-1.41)	0.005
24 mo	0.46	0.45	0.99 (0.85-1.15)	0.887
36 mo	0.52	0.54	1.08 (0.94-1.25)	0.290
ICC				
Control			0.019 (0.010-0.035)	
Intervention			0.017 (0.010-0.029)	
LR test				0.016
**Any prompt treatment (n = 18 753)**				
Overall†	0.59	0.61	1.12 (1.00-1.25)	0.054
Time point‡				
12 mo	0.64	0.69	1.24 (1.08-1.42)	0.003
24 mo	0.55	0.55	0.98 (0.85-1.13)	0.792
36 mo	0.56	0.59	1.13 (0.98-1.30)	0.100
ICC				
Control			0.016 (0.008-0.032)	
Intervention			0.013 (0.007-0.025)	
LR test				0.009
**Health sector evaluation (n = 20 088)**				
Overall†	0.62	0.64	1.12 (0.99-1.26)	0.072
Time point‡				
12 mo	0.63	0.67	1.19 (1.03-1.38)	0.016
24 mo	0.65	0.66	1.06 (0.91-1.22)	0.463
36 mo	0.57	0.60	1.10 (0.95-1.27)	0.216
ICC				
Control			0.020 (0.011-0.036)	
Intervention			0.019 (0.011-0.033)	
LR test				0.232
**Any care (N = 20 104)**				
Overall†	0.69	0.72	1.15 (1.03-1.28)	0.017
Time point‡				
12 mo	0.70	0.74	1.20 (1.04-1.38)	0.010
24 mo	0.73	0.75	1.07 (0.92-1.23)	0.386
36 mo	0.63	0.66	1.17 (1.01-1.34)	0.032
ICC				
Control			0.017 (0.009-0.032)	
Intervention			0.014 (0.007-0.027)	
LR test				0.282
**Recommended case management (n = 14 613)**				
Overall†	0.41	0.42	1.08 (0.96-1.21)	0.208
Time point‡				
12 mo	0.46	0.47	1.07 (0.92-1.25)	0.399
24 mo	0.39	0.41	1.14 (0.97-1.34)	0.109
36 mo	0.38	0.38	1.02 (0.87-1.20)	0.812
ICC				
Control			0.010 (0.003-0.028)	
Intervention			0.008 (0.003-0.022)	
LR test				0.5361
**Prompt, recommended case management (n = 14 612)**				
Overall†	0.36	0.37	1.09 (0.97-1.22)	0.164
Time point‡				
12 mo	0.42	0.44	1.08 (0.93-1.26)	0.332
24 mo	0.31	0.34	1.21 (1.03-1.42)	0.024
36 mo	0.33	0.33	0.98 (0.83-1.16)	0.787
ICC				
Control			0.008 (0.023- 0.025)	
Intervention			0.011 (0.005- 0.026)	
LR test				0.1106

AOR_CS_ – cluster-specific adjusted odds ratio, LR – likelihood ratio, ARC – absolute risk of events in the control arm, ARI – absolute risk of events in the intervention arm, C – control clusters, CI – confidence interval, I – intervention clusters, ICC – intracluster correlation coefficient, mo – months

*Two regression models are presented here: regression model 1 controlled for the time effect *t =* 1, 2, 3, to estimate the intervention effect during the three-year follow-up period overall. Regression model 2 included the interaction term *η_i_t* that estimated the intervention effect at each time point. The likelihood ratio test corresponds to the interaction term in model 2. Adjusted models controlled for child’s age (0-11, 12-23, 24-35, 36-59 mo) and sex; baseline cluster-level summary of the outcome; baseline cluster-level summary of household wealth (quintiles), mother’s decision-making power (any, none), and mother’s mobility (none, dependent mobility, independent mobility), which were deemed imbalanced at baseline and likely risk factors; PHC catchment area and cluster distance to PHC (coded as a continuous variable in the models for prompt treatment within the health sector, any prompt treatment, prompt, recommended case management, and pneumonia where the relationship with distance was linear, and otherwise coded as a dichotomous variable using a five-kilometre cut-off), which were the variables on which randomisation was stratified; and symptom (fever, diarrhoea with no blood, cough with fast breathing, combination), only for recommended case management outcomes.

†Regression model 1.

‡Regression model 2.

The results suggested no differential effect by time point for health sector evaluation and any care, although the largest effects were seen at 12 months (**Table 2**). During the three-year period overall, the odds of receiving any health sector evaluation was 12% higher in intervention compared to control clusters (AOR_CS_ = 1.12; 95% CI = 0.99, 1.26, *P* = 0.072), corresponding to an absolute difference of 2.5 percentage points (ARD = 0.025; 95% CI = -0.002, 0.052). Results were similar for any care. There was no evidence of an effect on recommended case management or prompt, recommended case management. We did not find statistical evidence for effect modification by cluster size, distance to PHC, or household wealth. However, estimated magnitudes suggest that the intervention may have been more effective in improving prompt treatment within the health sector (**Table 3**) and access to care across outcomes and time points (Tables S9-S10 in the **Online Supplementary Document**) in smaller, more remote clusters, and in the poorest households.

**Table 3:** Heterogeneous treatment effects by cluster population size, cluster distance to nearest PHC, and household wealth on the primary outcome, prompt treatment within the health sector, during the three-year trial period overall*

	ARC	ARI	C vs I, AOR_CS_ (95% CI)	*P*-value
**Cluster distance to PHC**				
≤5.0 km	0.54	0.55	1.01 (0.84-1.22)	0.918
>5.0 km	0.50	0.54	1.18 (1.01-1.38)	0.039
LR test				0.2193
**Cluster population size**				
<700 people	0.53	0.57	1.18 (0.99-1.41)	0.072
≥700	0.51	0.53	1.07 (0.91-1.24)	0.419
LR test				0.4132
**Household wealth†**				
Less poor	0.53	0.55	1.08 (0.95-1.22)	0.243
Poorest	0.49	0.54	1.23 (1.03-1.46)	0.022
LR test				0.1000

PHC – public health centre, LR – likelihood ratio, AOR_CS_ – cluster-specific adjusted odds ratio, ARC – absolute risk of events in the control arm, ARI – absolute risk of events in the intervention arm, C – control clusters, CI – confidence interval, I – intervention clusters

*We ran three separate models, one for each of the predefined effect modifiers that included an interaction term between treatment arm and the modifier. We report the results of the LR tests for interaction between arm and modifier in each model. All models controlled for the same covariates as the main model for overall effects during the three-year trial period; we removed the baseline cluster-level summary of wealth in the models that assessed heterogeneous effects by this variable at the household level.

†For 20% of sick child-year observations included in the analysis, their household wealth was measured during the follow-up period rather than at baseline. The co-intervention in both arms to remove user fees could have influenced household wealth in the follow-up period.

During the trial, 47% of sick child-year observations met the per-protocol definition (at least two CHW home visits in the preceding month) in the intervention arm, while 78% met the definition (no CHW home visits in the preceding month) in the control arm (Table S11 in the **Online Supplementary Document**). The proportion that met the per-protocol definition waned over time in the intervention arm (53% at 12, 49% at 24, 39% at 36 months) and increased in the control arm (72% at 12, 81% at 24, 83% at 36 months). Restricted to the per-protocol subgroup, the intervention effect on prompt treatment within the health sector increased to 45% higher odds at 12 months (AOR_CS_ = 1.43; 95% CI = -1.21, 1.69, *P* < 0.001) and 22% over the three years (AOR_CS_ = 1.22; 95% CI = -1.06, 1.40, *P* = 0.005), compared to control clusters (**Table 4**). Health sector evaluation odds were 29% higher in the intervention compared to control clusters over the three years (AOR_CS_ = 1.29; 95% CI = -1.12, 1.48, *P* < 0.001). Per-protocol analyses also yielded significant effects over the three years on recommended case management (AOR_CS_ = 1.20; 95% CI = -1.06, 1.37, *P* = 0.005). Results were consistent whether or not we excluded control arm observations that did not meet per-protocol (Table S12 in the **Online Supplementary Document**).

**Table 4:** Per-protocol subgroup estimates for the primary and secondary health care utilisation outcomes, excluding observations in the intervention arm that did not receive at least two CHW home visits in the month preceding the survey, during the three-year trial period overall and at each follow-up time point*

Outcome	ARC	ARI	C vs I, AOR_CS_ (95% CI)	*P*-value
**Prompt treatment within the health sector (n = 13 500)**				
Overall†	0.52	0.57	1.22 (1.06-1.40)	0.005
Time point‡				
12 mo	0.58	0.66	1.43 (1.21-1.69)	<0.001
24 mo	0.46	0.48	1.07 (0.89-1.28)	0.453
36 mo	0.52	0.55	1.13 (0.94-1.35)	0.184
LR test				0.0036
**Any prompt treatment (n = 13 493)**				
Overall†	0.59	0.64	1.26 (1.09-1.44)	0.001
Time point‡				
12 mo	0.64	0.72	1.43 (1.21-1.69)	<0.001
24 mo	0.55	0.58	1.10 (0.92-1.32)	0.315
36 mo	0.56	0.61	1.23 (1.03-1.47)	0.023
LR test				0.0205
**Health sector evaluation (n = 14 518)**				
Overall†	0.62	0.68	1.29 (1.12-1.48)	<0.001
Time point‡				
12 mo	0.63	0.70	1.38 (1.17-1.65)	<0.001
24 mo	0.65	0.71	1.30 (1.09-1.55)	0.003
36 mo	0.58	0.61	1.17 (0.97-1.39)	0.094
LR test				0.1736
**Any care (n = 14 527)**				
Overall†	0.69	0.75	1.35 (1.17-1.55)	<0.001
Time point‡				
12 mo	0.70	0.76	1.37 (1.16-1.63)	<0.001
24 mo	0.73	0.79	1.35 (1.13-1.62)	0.001
36 mo	0.63	0.69	1.31 (1.10-1.57)	0.003
LR test				0.8945
**Recommended case management (n = 10 569)**				
Overall†	0.42	0.45	1.20 (1.06-1.37)	0.005
Time point‡				
12 mo	0.46	0.49	1.19 (0.99-1.42)	0.061
24 mo	0.39	0.45	1.35 (1.12-1.63)	0.001
36 mo	0.38	0.39	1.06 (0.86-1.30)	0.592
LR test				0.1435
**Prompt, recommended case management (n = 10 569)**				
Overall†	0.36	0.39	1.15 (1.01-1.32)	0.040
Time point‡				
12 mo	0.43	0.46	1.19 (0.99-1.43)	0.062
24 mo	0.31	0.36	1.26 (1.04-1.53)	0.017
36 mo	0.33	0.33	0.99 (0.80-1.22)	0.890
LR test				0.1280

AOR_CS_ – cluster-specific adjusted odds ratio, ARC – absolute risk of events in the control arm, ARI – absolute risk of events in the intervention arm, C – control clusters, CI – confidence interval, I – intervention clusters, ICC – intracluster correlation coefficient, LR – likelihood ratio, mo – months

*Two regression models are presented here: regression model 1 controlled for the time effect *t =* 1, 2, 3, to estimate the intervention effect during the three-year follow-up period overall. Regression model 2 included the interaction term *η_i_t* that estimated the intervention effect at each time point. The likelihood ratio test corresponds to the interaction term in model 2. Adjusted models controlled for child’s age (0-11, 12-23, 24-35, 36-59 mo) and sex; baseline cluster-level summary of the outcome; baseline cluster-level summary of household wealth (quintiles), mother’s decision-making power (any, none), and mother’s mobility (none, dependent mobility, independent mobility), which were deemed imbalanced at baseline and likely risk factors; PHC catchment area and cluster distance to PHC (coded as a continuous variable in the models for prompt treatment within the health sector, any prompt treatment, prompt, recommended case management, and pneumonia where the relationship with distance was linear, and otherwise coded as a dichotomous variable using a five-kilometre cut-off), which were the variables on which randomisation was stratified; and symptom (fever, diarrhoea with no blood, cough with fast breathing, combination), only for recommended case management outcomes.

†Regression model 1.

‡Regression model 2.

Finally, infectious disease prevalence increased in both arms compared to baseline, two-fold for cough and suspected pneumonia (Figure S3 in the **Online Supplementary Document**). There was no intervention effect on any disease prevalence during the three years overall, although the odds of cough and suspected pneumonia were 1.16 times (95% CI = 1.04, 1.30) or 2.2 percentage points and 1.22 times (95% CI = 1.07, 1.40) or 1.6 percentage points higher, respectively, at 12 months in the intervention compared to control clusters, with consistent results in the per-protocol analyses (Table S13, S16, and S17 in the **Online Supplementary Document**).

## DISCUSSION

Early access to health sector treatment more than doubled for sick children when study communities received care from professional CHWs and upgraded primary care clinics without user fees. In 2018, the Mali DHS found that only 21% and 55% of children under five with fever in the Mopti region received any prompt treatment and any care, respectively [32]. In that same year, our 12-month survey found that any prompt treatment and any care reached two-thirds or more of all sick children under five in the trial area of Mopti. Health care utilisation peaked at 12 months and waned over time, and many sick children still did not receive prompt, health sector, or recommended care. Nevertheless, this overall improvement in child access to care is remarkable in the context of the performance of large-scale iCCM programme [15-17] and the armed conflict that emerged after 12 months in the trial area, imposing challenges to delivering and receiving services. It is in this redesigned health system context that the results between arms on the effects of proactive CHW home visits should be interpreted.

Proactive CHW service delivery improved early health sector treatment further, compared to the fixed approach, after 12 months, but not after 24 or 36 months of implementation. These findings suggest that home visits were most important during the first year after launching the redesigned CHW-led health system, possibly by mobilising care-seeking, reinforcing the importance of prompt treatment, or building trust in the health system. After more than a year of experiencing accessible, high-quality care without fees, control communities with fixed CHWs may have themselves mobilised, adopted rapid care-seeking, and gained trust in the system, though not as quickly. There was some evidence that, over all three years, proactive CHW service delivery improved access to health sector evaluation and any care, suggesting that home visits may have helped to overcome persistent indirect cost, distance, or social barriers to care, even where fixed CHW services were available without fees. Subgroup estimates suggested that proactive home visits may improve child access to care best in smaller communities, where a CHW can achieve greater home visit coverage, in those farther from a PHC, where utilisation was lowest at baseline [27], and in the poorest households, by overcoming indirect costs to even frontline services or women’s limited resources to make health care decisions. Although these subgroup results should be interpreted with caution, they may contribute to the evidence that home visits enhance equity benefits of CHW programmes, along with the important equity impacts of free, proximal, quality service provision [33,34].

For maternal health care, our analysis of other secondary trial endpoints (reported elsewhere) [35] found that proactive CHW home visits increased the likelihood of first trimester antenatal care (ANC) by 11% (risk ratio (RR) = 1.11; 95% CI = 1.02, 1.19) and of four or more ANC visits by 25% (RR = 1.25; 95% CI = 1.08, 1.43), but had no effect on institutional delivery (RR=1.06; 95% CI = 0.91, 1.20). Across trial arms relative to baseline, any ANC attendance increased by 83% (RR = 1.83; 95% CI = 1.78, 1.86), first trimester ANC by 15% (RR = 1.15; 95% CI = 1.06, 1.25), four or more ANC visits by 2.6 times (RR = 2.59; 95% CI = 2.28, 2.91), and institutional delivery by 54% (RR = 1.54; 95% CI = 1.41, 1.66) [35]. These maternal care results are consistent with the child health care utilisation results insomuch that the bulk of the improvements occurred across both arms, with the proactive service delivery intervention yielding modest incremental benefits, which are nonetheless important for achieving timely, universal health coverage.

CHW adherence to the proactive workflow protocol, as reported at survey time points by respondents, reached only half of sick children in the intervention arm and waned over time. This could be intervention fatigue or the conflict making the proactive workflow difficult to deliver. This likely biased ITT intervention effect estimates towards the null, as per-protocol subgroup analyses showed stronger magnitudes and significance of effects across children’s utilisation outcomes at 12 months and during the trial overall. These findings suggest that had households in the intervention arm received a proactive CHW home visit at least once every two weeks throughout the trial period, home visits may have had more effect on children’s health care utilisation.

The proactive service delivery intervention effects found in this trial should be understood within the context of the co-interventions in both trial arms, including user fee removal, professional CHWs, and upgraded primary care clinics. Proactive CHW home visits’ effects may be different in other health system or social contexts. Our forthcoming process evaluation paper used mixed methods to elucidate the implementation, mechanisms, and context of the proactive home visits and co-interventions in both arms and to help to explain these trial outcome results (unpublished data).

Child morbidity, measured as disease prevalence, did not decrease over time or more so in the intervention arm as we expected it to. Rather, reported prevalence of all four illnesses increased during the trial period compared to baseline (descriptive), and cough and suspected pneumonia increased statistically at 12 months in intervention compared to control clusters. These increases could reflect mothers’ improved illness recognition given CHW care and, additionally, home visits. Mothers who received routine counselling during home visits on disease prevention, illness recognition, and rapid care-seeking may have been more likely during the first year than their control arm counterparts to recognise cough as an illness and fast breathing as an alert, and thus report it during a survey. Our study did not measure progression or severity of disease, which may link health care utilisation to survival in the pathway of change, and this is a limitation. In Ghana, home visits by volunteer CHWs focusing on health education, but who also tested febrile children for malaria and treated childhood diarrhoea with ORS, had no effect on the prevalence of these illnesses (primary outcomes) or case detection/management, compared to no volunteer CHWs [36]. Although our trial also did not find expected reductions in the prevalence of these illnesses, we did find that recommended case management of iCCM illnesses doubled during our intervention of paid, professional CHWs, compared to baseline.

With its randomised design, large number of clusters, and rigorous, baseline, and repeated outcome measurement, this trial addressed common risks of bias found in studies in this domain [24]. Contamination between arms is an important concern and could have occurred because CHWs did not always adhere to their workflow protocol; co-interventions may have triggered mechanistic pathways of proactive home visits, such as supervisor house calls without the CHW or community mobilisation by village chiefs; or study participants could have migrated between clusters. The armed conflict that emerged led to devastating death and displacement, contributing to our loss to follow-up, but all clusters and participants contributed data to the analysis. We also had missing treatment data for some sick children at 24 months, which is an important limitation, but our complete-case analysis results were robust to multiple imputation.

## CONCLUSIONS

This analysis showed that proactive CHW service delivery can improve the timeliness of children’s curative treatment within the first year of implementing a redesigned CHW-led health system, and may increase sick children’s health care utilisation relative to a fixed CHW approach. In the context of user fee removal, professional CHWs, and upgraded primary care clinics, proactive CHW home visits yielded modest improvements in access to child and maternal health care. While policy-makers, public health practitioners, and clinicians may consider proactive home visits to be a low-cost intervention for optimising CHW programmes, the UHC and equity impact they seek will be primarily driven by health system enablers, such as user fee removal, professional CHWs, and reinforced primary care clinics.

## Additional material


Online Supplementary Document
