# Supplementary material for: Effects of proactive vs fixed community health care delivery on child health and access to care: a cluster randomised trial secondary endpoint analysis
Source: J Glob Health. 2023 Apr 21;13:04047. doi: 10.7189/jogh.13.04047 (PMC10122537; doi:10.7189/jogh.13.04047)
Supplement: Online Supplementary Document [file jogh-13-04047-s001.pdf]

Online Supplementary Document

Supplementary Tables/Figures

Figure S1: Timeline cluster graphical tool

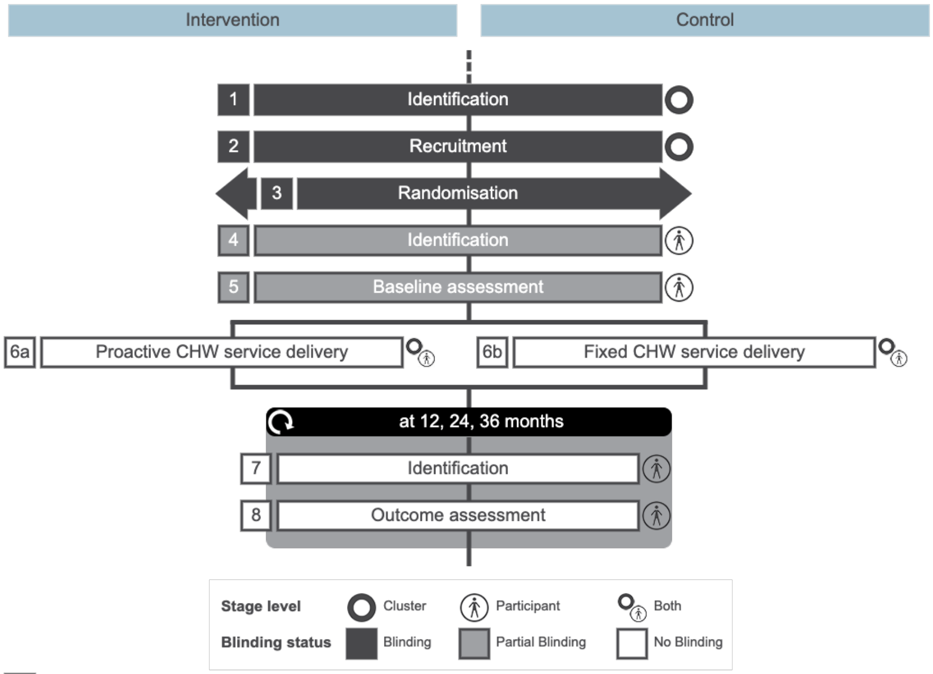

1 Identification
Seven contiguous PHC catchment areas (of 22) in the Bankass health district are chosen by the implementing organization's leadership and trial PIs in partnership with the Malian Ministry of Health and Social Affairs (MSDS), based on high under-five mortality and low health-care utilization, few concurrent health interventions, and availability of local health authorities to collaborate.

2 Recruitment
The intervention implementation team holds public consultation meetings with community representatives from each PHC catchment area, including village chiefs and their advisors, leaders of women's and youth associations, religious leaders, mayors and other politico-administrative officials, PHC directors and community health associations. Verbal permission to conduct the trial is obtained at these meetings.

3 Randomisation
Based on an initial geo-mapping, villages and hamlets one kilometre or less apart are grouped into clusters. Clusters are randomised in a 1:1 allocation and assigned to treatment arms by a trial statistician not involved in implementation or the identification of participants. Randomisation is stratified on PHC catchment area and distance to nearest PHC. In total, there are 21 strata. Each of the seven catchment areas has three strata: one for the cluster where the PHC is located, one for clusters within five kilometres from the PHC, and one for clusters beyond five kilometres.

4 Identification
The entire cluster is studied. An initial enumeration identifies all family compounds in each cluster. Four-digit identifiers are assigned and displayed at the entrance to each family compound, starting with the village chief (0001). All permanent residents are enrolled in a population census at a subsequent field visit, when baseline assessors collect household roster and socio-economic information within each family compound. Open Data Kit is programmed to identify, in real-time, women in the household roster to be routed through an eligibility module, where screening against the inclusion criteria and consent to participate in the women's survey is obtained. Overall study objectives and design, including the randomisation procedure, are discussed with assessors during their training and with participants during informed consent. Although there is no explicit disclosure of allocation to either actor, it is not possible to know for sure whether allocation concealment is achieved at this stage.

5 Baseline assessment
Reproductive, maternal, newborn, and child health service utilisation outcomes are assessed at baseline with the women's survey that collects self-reported data. Although there is no explicit disclosure of allocation to either assessors or participants, it is not possible to know for sure whether allocation concealment is achieved at this stage.

6a Intervention delivery
Comprehensive community-based primary care services are provided by professionalised CHWs in every cluster, at a CHW:population ratio of approximately 1:700. Small clusters that have a population of less than 200 people and a neighbouring cluster allocated to the same treatment arm located less than three kilometres away, and without other geographical or language barriers separating the two, are assigned the same CHW (N=5 intervention cluster pairs). In the intervention arm, CHWs deliver services using a proactive approach: CHWs conduct door-to-door proactive case-finding home visits for at least two hours per day, six days per week, with the goal of visiting every household at least twice per month. Given the nature of the intervention, blinding of participants and study implementers to allocation is not possible. Dedicated CHW supervisors provide individual, monthly supervision to CHWs in both arms. PHCs that serve both arms are reinforced in physical infrastructure, equipment, stocks, and supplies, as well as in qualified personnel and training in primary care. User fees are removed for CHW and PHC care, and at higher levels of care for referred patients.

6b Comparison delivery
Comprehensive community-based primary care services are provided by professionalised CHWs in every cluster, at a CHW:population ratio of approximately 1:700. Small clusters that have a population of less than 200 people and a neighbouring cluster allocated to the same treatment arm located less than three kilometres away, and without other geographical or language barriers separating the two, are assigned the same CHW (N=6 control cluster pairs). In the control arm, CHWs deliver services using the conventional, passive approach: CHWs provide care at community health sites for at least four hours per day, six days per week, to care-seeking patients. Given the nature of the intervention, blinding of participants and study implementers to allocation is not possible. Dedicated CHW supervisors provide individual, monthly supervision to CHWs in both arms. PHCs that serve both arms are reinforced in physical infrastructure, equipment, stocks, and supplies, as well as in qualified personnel and training in primary care. User fees are removed for CHW and PHC care, and at higher levels of care for referred patients.

7 Identification
New entrants to the cluster are studied. The population census is updated at each annual follow-up data collection, capturing births and deaths, and permanent in- and out-migration to the study area. Newly eligible women, including those who have turned 15 years old or have married into the area in the interim period, are identified and passed through the screening module. Women who were enrolled in a previous annual survey are asked to participate in follow-up surveys even if they have aged beyond 49 years. Given the nature of the ongoing intervention, blinding of assessors and participants to allocation is not possible.

8 Outcome assessment
The trial primary endpoint, under-five mortality, is assessed with the self-reported lifetime birth history in the women's survey at each follow-up. Child health and service utilisation outcomes are assessed with mother's two-week recall for each of her co-residing, living children under five at each survey time-point. Given the nature of the ongoing intervention, blinding of assessors and participants to allocation is not possible. Statisticians are blind to allocation throughout the trial (after step 3) until the dataset is fully cleaned and locked and the randomisation assignment is provided by the DSMB.

Figure S2: Trial profile

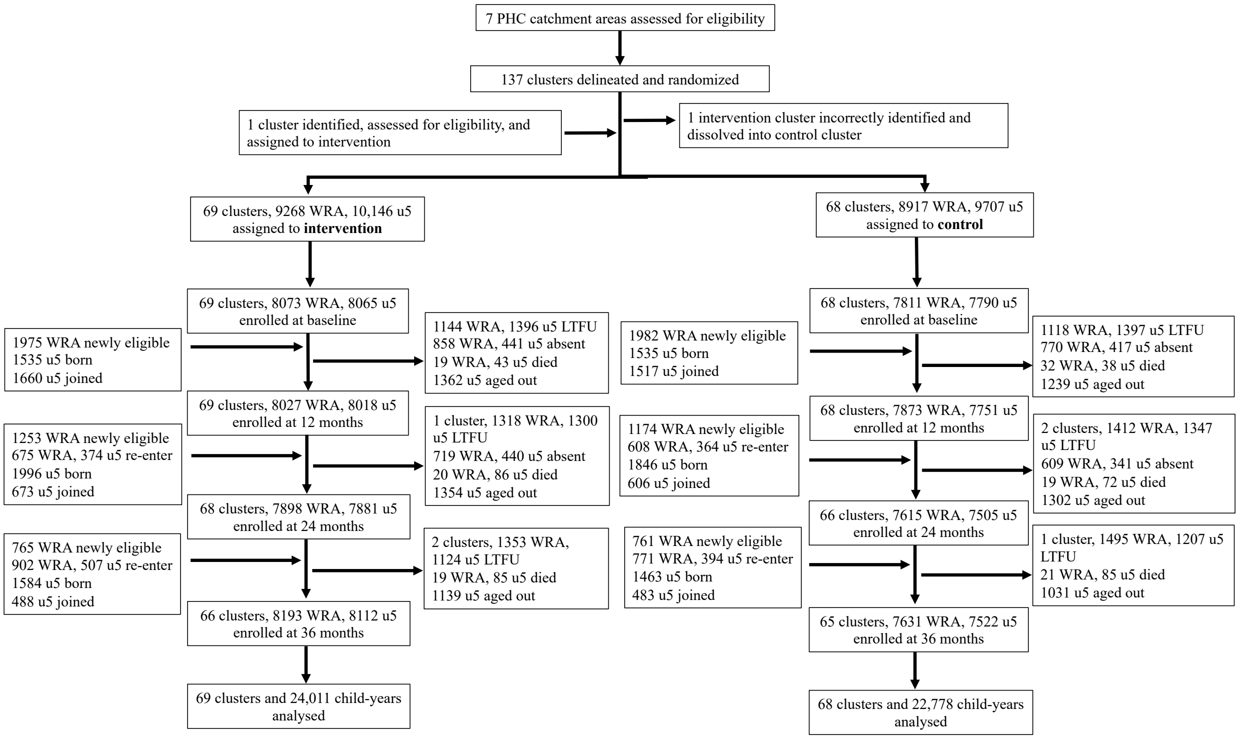

Notes: Following randomisation, an additional eligible cluster was identified and assigned to the intervention arm, the same arm as its nearest neighbour. A neighbourhood within a control arm cluster was wrongly randomised to the intervention arm as a separate cluster and was later combined/reassigned. Both changes were made to reduce the potential for contamination. Numbers for women of reproductive age (WRA) assigned to treatment arms come from the screening module at baseline, to which women were routed from the population census based on their age and screened against the other eligibility criteria and asked to participate. Numbers for children under five years of age (u5) assigned to treatment arms come from the baseline population census. Numbers enrolled at each time-point are derived using the women's survey panel for WRA and the child morbidity panel for u5. Reasons for inclusion and exclusion between survey waves for children under five were derived using data from women's lifetime birth histories available for mothers surveyed during the follow-up period, and imputed based on child's age if missing data from the birth histories. Absent and re-enter refer to participants who skipped one or two survey waves and reappear in the cohort. Six entire clusters were lost to follow-up (LTFU) during the trial due to the armed conflict: four were destroyed and two were inaccessible to the survey team.

Table S1: Characteristics of all children under five years of age with any symptom at each time-point

	Baseline				12 months				24 months				36 months			
	Intervention N=69 clusters 4411 HHs 5296 mothers 8065 children		Control N=68 clusters 4364 HHs 5197 mothers 7790 children		Intervention N=69 clusters 4518 HHs 5375 mothers 8018 children		Control N=68 clusters 4401 HHs 5217 mothers 7751 children		Intervention N=67 clusters 4389 HHs 5223 mothers 7881 children		Control N=66 clusters 4180 HHs 4981 mothers 7505 children		Intervention N=66 clusters 4522 HHs 5478 mothers 8112 children		Control N=65 clusters 4158 HHs 5042 mothers 7552 children	
	N	%	N	%	N	%	N	%	N	%	N	%	N	%	N	%
Household-level characteristics																
Household size																
Median/IQR	8	5, 11	7	5, 10	8	5, 12	8	5, 11	8	5, 12	8	5, 11	8	5, 12	8	5, 12
Household wealth quintile																
Poorest	1243	15.3	995	12.8	1358	16.9	1256	16.2	1431	18.2	1260	16.8	1619	20.0	1418	18.9
Poor	1465	18.2	1560	20.0	1440	18.0	1565	20.2	1419	18.0	1533	20.4	1520	18.7	1526	20.3
Middle	1573	19.5	1587	20.4	1627	20.3	1570	20.3	1595	20.2	1479	19.7	1621	20.0	1446	19.2
Rich	1773	22.0	1791	23.0	1744	21.8	1661	21.4	1614	20.5	1626	21.7	1589	19.6	1589	21.1
Richest	1952	24.2	1786	22.9	1801	22.5	1657	21.4	1788	22.7	1584	21.1	1760	21.7	1543	20.5
Missing	68	0.8	69	0.89	48	0.6	42	0.5	34	0.4	23	0.3	3	0.0	0	0
Mother-level characteristics																
Marital status																
Not married	89	1.1	79	1.0	72	0.9	73	0.9	66	0.8	71	1.0	97	1.2	67	0.9
Polygynous	3417	42.4	3363	43.2	3568	44.5	3352	43.3	3566	45.3	3376	45.0	3819	47.1	3547	47.2
Monogamous	4556	56.5	4339	55.7	4376	54.6	4324	55.8	4246	53.9	4055	54.0	4193	51.7	3908	52.0
Missing	3	0.0	9	0.1	2	0.0	2	0.0	3	0.0	3	0.0	3	0.0	0	0
Parity (reported live births)																
Median/IQR	5	3, 8	5	3, 8	4	3, 6	4	3, 6	5	3, 7	4	3, 7	5	3, 7	5	3, 7
1-2	1280	15.9	1277	16.4	1797	22.4	1777	22.9	1712	21.7	1613	21.5	1757	21.7	1649	21.9
3-4	1889	23.4	1926	24.7	2382	29.7	2428	31.3	2142	27.2	2162	28.8	2241	27.6	2098	27.9
5-6	1522	18.9	1485	19.1	1985	24.8	1884	24.3	1804	22.9	1695	22.6	1953	24.1	1858	24.7
7+	2663	33.0	2357	30.3	1854	23.1	1662	21.4	2223	28.2	2035	27.1	2161	26.6	1917	25.5
Missing	711	8.8	745	9.6	0	0	0	0	0	0	0	0	0	0	0	0
Mother's education																
None	7503	93.0	7151	91.8	6829	85.2	6528	84.2	6677	84.7	6269	83.5	6778	83.6	6260	83.2
Any	556	6.9	633	8.1	566	7.1	690	8.9	677	8.6	809	10.8	794	9.8	881	11.7
Missing	6	0.1	6	0.1	623	7.8	533	6.9	527	6.7	427	5.7	540	6.7	381	5.1

Mother participates in paid labour																
No	6973	86.5	6716	86.2	6392	79.7	6247	80.6	6673	84.7	6250	83.3	7009	86.4	6633	88.2
Yes	1067	13.2	1055	13.5	1602	20.0	1471	19.0	1204	15.3	1253	16.7	1103	13.6	884	11.8
Missing	25	0.3	19	0.2	24	0.3	33	0.4	4	0.1	2	0.0	0	0	5	0.1
Mother's mobility*																
No place	2686	33.3	2614	33.6	2359	29.4	2305	29.7	2290	29.1	2190	29.2	2276	28.1	2181	29.0
Any place but none alone	1336	16.6	1431	18.4	1304	16.3	1392	18.0	1260	16.0	1333	17.8	1360	16.8	1393	18.5
Any place alone	4028	49.9	3734	47.9	3724	46.5	3518	45.4	3708	47.1	3490	46.5	3777	46.6	3451	45.9
Missing	15	0.2	11	0.1	631	7.9	536	6.9	623	7.9	492	6.6	699	8.6	497	6.6
Mother's decision-making																
None	5789	71.8	5813	74.6	5620	70.1	5399	69.7	5524	70	5208	69.4	5814	71.7	5239	69.7
Any	2267	28.1	1972	25.3	2397	29.9	2349	30.3	2339	29.7	2284	30.4	2294	28.3	2279	30.3
Missing	9	0.1	5	0.1	1	0.0	3	0.0	18	0.2	13	0.2	4	0.1	4	0.1
Child-level characteristics																
Sex																
Male	4080	50.6	3954	50.8	4049	50.5	3960	51.1	3934	49.9	3805	50.7	4041	49.8	3846	51.1
Female	3985	49.4	3836	49.2	3969	49.5	3791	48.9	3947	50.1	3700	49.3	4071	50.2	3676	48.9
Age (in months)																
Median/IQR	29	13,44	28	13,43	28	15,44	28	15,44	28	14,42	28	14,42	29	14,43	29	14,43
0-11	1703	21.1	1639	21.0	1467	18.3	1460	18.8	1695	21.5	1569	20.9	1578	19.5	1446	19.2
12-23	1582	19.6	1560	20.0	1805	22.5	1757	22.7	1614	20.5	1588	21.2	1748	21.6	1599	21.3
24-35	1586	19.7	1552	19.9	1567	19.5	1531	19.8	1675	21.3	1614	21.5	1640	20.2	1547	20.6
36-59	3194	39.6	3039	39.0	3179	39.7	3003	38.7	2897	36.8	2734	36.4	3146	38.8	2930	39.0

Notes: *Women's mobility is coded categorically based on them reporting having ever been to the marketplace, health centre, women's group, or outside the village (never been to any, been to some or all but never alone, been to at least one alone). HHs=households.

Table S2: Characteristics of sick children under five years of age with any symptom at each time-point

	Baseline				12 months				24 months				36 months			
	Intervention N=67 clusters 2097 HHs 2343 mothers 2830 children		Control N=68 clusters 1893 HHs 2095 mothers 2512 children		Intervention N=69 clusters 2715 HHs 3023 mothers 3700 children		Control N=66 clusters 2507 HHs 2789 mothers 3339 children		Intervention N=67 clusters 2567 HHs 2865 mothers 3425 children		Control N=66 clusters 2435 HHs 2712 mothers 3253 children		Intervention N=66 clusters 2518 HHs 2800 mothers 3316 children		Control N=65 clusters 2343 HHs 2615 mothers 3072 children	
	N	%	N	%	N	%	N	%	N	%	N	%	N	%	N	%
Household-level characteristics																
Household size																
Median/IQR	7	5, 10	7	5, 10	8	5, 11	8	5, 11	8	5, 12	8	5, 11	8	5, 12	8	5, 11
Household wealth quintile																
Poorest	494	17.5	351	14.0	652	17.6	533	16.0	611	17.8	532	16.4	663	20.0	575	18.7
Poor	546	19.3	507	20.2	662	17.9	659	19.7	626	18.3	654	20.1	592	17.9	624	20.3
Middle	553	19.5	505	20.1	725	19.6	687	20.6	743	21.7	648	19.9	662	20.0	597	19.4
Rich	576	20.4	545	21.7	830	22.4	761	22.8	669	19.5	702	21.6	683	20.6	654	21.3
Richest	628	22.2	577	23.0	808	21.8	690	20.7	762	22.3	705	21.7	714	21.5	622	20.3
Missing	33	1.2	27	1.1	23	0.6	9	0.3	14	0.4	12	0.4	2	0.1	575	18.7
Mother-level characteristics																
Marital status																
Not married	32	1.1	29	1.2	43	1.2	29	0.9	29	0.9	26	0.8	38	1.2	32	1.0
Polygynous	1138	40.2	1074	42.8	1651	44.6	1474	44.1	1552	45.3	1435	44.1	1565	47.2	1443	47.0
Monogamous	1659	58.6	1406	56.0	2005	54.2	1835	55.0	1843	53.8	1790	55.0	1712	51.6	1597	52.0
Missing	1	0.1	3	0.1	1	0.0	1	0.0	1	0.0	2	0.1	1	0.0	32	1.0
Parity (reported live births)																
Median/IQR	5	3, 8	5	3, 7	4	3, 6	4	3, 6	5	3, 7	5	3, 7	5	3, 7	4	3, 7
1-2	486	17.2	423	16.8	852	23.0	750	22.5	779	22.7	741	22.8	758	22.9	728	23.7
3-4	649	22.9	601	23.9	1071	29.0	998	29.9	873	25.5	884	27.2	871	26.3	812	26.4
5-6	509	18.0	477	19.0	885	23.9	797	23.9	791	23.1	708	21.8	792	23.9	738	24.0
7+	916	32.4	792	31.5	892	24.1	794	23.8	982	28.7	920	28.3	895	27.0	794	25.9
Missing	270	9.5	219	8.7	0	0	0	0	0	0	0	0	0	0	0	0
Mother's education																
None	2606	92.1	2300	91.6	3165	85.5	2842	85.1	2894	84.5	2760	84.8	2742	82.7	2586	84.2
Any	223	7.9	211	8.4	263	7.1	266	8.0	307	9.0	327	10.1	343	10.3	349	11.4
Missing	1	0.0	1	0.0	272	7.4	231	6.9	224	6.5	166	5.1	231	7.0	137	4.5

Mother participates in paid labour																
No	2363	83.5	2053	81.7	2889	78.1	2660	79.7	2861	83.5	2676	82.3	2777	83.8	2670	86.9
Yes	456	16.1	454	18.1	802	21.7	663	19.9	561	16.4	577	17.7	539	16.3	399	13.0
Missing	11	0.4	5	0.2	9	0.2	16	0.5	3	0.1	0	0	0	0	3	0.1
Mother's mobility*																
No place	619	21.9	642	25.6	982	26.5	962	28.8	977	28.5	963	29.6	924	27.9	898	29.2
Any place but none alone	587	20.7	515	20.5	627	17.0	547	16.4	526	15.4	564	17.3	554	16.7	557	18.1
Any place alone	1620	57.2	1354	53.9	1818	49.1	1595	47.8	1651	48.2	1528	47.0	1548	46.7	1425	46.4
Missing	4	0.1	1	0.0	273	7.4	235	7.0	271	7.9	198	6.1	290	8.8	192	6.3
Mother's decision-making																
None	1851	65.4	1772	70.5	2489	67.3	2257	67.6	2385	69.6	2233	68.6	2319	69.9	2121	69.0
Any	976	34.5	738	29.4	1210	32.7	1079	32.3	1034	30.2	1015	31.2	996	30.0	950	30.9
Missing	3	0.1	2	0.1	1	0.0	3	0.1	6	0.2	5	0.2	1	0.0	1	0.0
Child-level characteristics																
Sex																
Male	1433	50.6	1255	50.0	1916	51.8	1753	52.5	1758	51.3	1660	51.0	1666	50.2	1575	51.3
Female	1397	49.4	1257	50.0	1784	48.2	1586	47.5	1667	48.7	1593	49.0	1650	49.8	1497	48.7
Age (in months)																
Median/IQR	29	15,43	27	14,42	28	15,44	27	15,42	27	15,40	26	14,40	27	14,41	28	14,41
0-11	521	18.4	464	18.5	694	18.8	628	18.8	711	20.8	677	20.8	641	19.3	574	18.7
12-23	599	21.2	573	22.8	868	23.5	805	24.1	776	22.7	773	23.8	805	24.3	727	23.7
24-35	587	20.7	554	22.1	718	19.4	685	20.5	770	22.5	697	21.4	710	21.4	674	21.9
36-59	1123	39.7	921	36.7	1420	38.4	1221	36.6	1168	34.1	1106	34.0	1160	35.0	1097	35.7

Notes: *Women's mobility is coded categorically based on them reporting having ever been to the marketplace, health centre, women's group, or outside the village (never been to any, been to some or all but never alone, been to at least one alone). HHs=households.

Table S3: Characteristics of sick children aged 3-59 months with iCCM illnesses at each time-point

	Baseline				12 months				24 months				36 months			
	Intervention N=67 clusters 1670 HHs 1831 mothers 2138 children		Control N=68 clusters 1456 HHs 1584 mothers 1826 children		Intervention N=69 clusters 2266 HHs 2475 mothers 2908 children		Control N=66 clusters 2020 HHs 2236 mothers 2579 children		Intervention N=67 clusters 2064 HHs 2268 mothers 2612 children		Control N=65 clusters 1940 HHs 2125 mothers 2462 children		Intervention N=66 clusters 1955 HHs 2145 mothers 2427 children		Control N=65 clusters 1859 HHs 2036 mothers 2290 children	
	N	%	N	%	N	%	N	%	N	%						
Household-level characteristics																
Household size																
Median/IQR	7	5, 10	7	5, 11	8	5, 11	8	5, 11	8	5, 12	8	5, 11	8	5, 12	8	5, 11
Household wealth quintile																
Poorest	374	17.5	269	14.7	504	17.3	419	16.3	464	17.8	384	15.6	485	20.0	423	18.5
Poor	416	19.5	354	19.4	528	18.2	521	20.2	437	16.7	489	19.9	430	17.7	465	20.3
Middle	430	20.1	354	19.4	568	19.5	531	20.6	583	22.3	497	20.2	483	19.9	450	19.7
Rich	424	19.8	422	23.1	656	22.6	572	22.2	507	19.4	543	22.1	490	20.2	477	20.8
Richest	470	22.0	410	22.5	635	21.8	532	20.6	608	23.3	541	22.0	538	22.2	475	20.7
Missing	24	1.1	17	0.9	17	0.6	4	0.2	13	0.5	8	0.3	1	0.0	0	0
Mother-level characteristics																
Marital status																
Not married	25	1.2	19	1.0	37	1.3	21	0.8	21	0.8	19	0.8	25	1.0	20	0.9
Polygynous	878	41.1	776	42.5	1314	45.2	1152	44.7	1208	46.3	1099	44.6	1161	47.8	1078	47.1
Monogamous	1234	57.7	1029	56.4	1556	53.5	1406	54.5	1382	52.9	1342	54.5	1241	51.1	1192	52.1
Missing	1	0.1	2	0.1	1	0.0	0	0	1	0.0	2	0.1	0	0	0	0
Parity (reported live births)																
Median/IQR	5	3, 8	5	3, 8	4	3, 6	4	3, 6	5	3, 7	5	3, 7	5	3, 7	5	3, 7
1-2	353	16.5	320	17.5	669	23.0	557	21.6	583	22.3	549	22.3	564	23.2	529	23.1
3-4	478	22.4	427	23.4	838	28.8	776	30.1	652	25.0	671	27.3	632	26.0	604	26.4
5-6	396	18.5	330	18.1	687	23.6	627	24.3	606	23.2	533	21.7	578	23.8	538	23.5
7+	703	32.9	593	32.5	714	24.6	619	24.0	771	29.5	709	28.8	653	26.9	619	27.0
Missing	208	9.7	156	8.5	0	0	0	0	0	0	0	0	0	0	0	0
Mother's education																
None	1974	92.3	1681	92.1	2507	86.2	2214	85.9	2211	84.7	2090	84.9	1998	82.3	1937	84.6
Any	164	7.7	144	7.9	199	6.8	193	7.5	223	8.5	249	10.1	260	10.7	250	10.9
Missing	0	0	1	0.1	202	7.0	172	6.7	178	6.8	123	5.0	169	7.0	103	4.5

Mother participates in paid labour																
No	1771	82.8	1492	81.7	2299	79.1	2061	79.9	2189	83.8	2016	81.9	2038	84.0	1994	87.1
Yes	359	16.8	330	18.1	600	20.6	507	19.7	420	16.1	446	18.1	389	16.0	296	12.9
Missing	8	0.4	4	0.2	9	0.3	11	0.4	3	0.1	0	0	0	0	0	0
Mother's mobility*																
No place	451	21.1	467	25.6	767	26.4	732	28.4	721	27.6	742	30.1	672	27.7	659	28.8
Any place but none alone	447	20.9	371	20.3	495	17.0	421	16.3	413	15.8	415	16.9	391	16.1	424	18.5
Any place alone	1237	57.9	988	54.1	1440	49.5	1251	48.5	1265	48.4	1157	47.0	1151	47.4	1063	46.4
Missing	3	0.1	0	0	206	7.1	175	6.8	213	8.2	148	6.0	213	8.8	144	6.3
Mother's decision-making																
None	1377	64.4	1270	69.6	1948	67.0	1721	66.7	1808	69.2	1672	67.9	1695	69.8	1563	68.3
Any	758	35.5	555	30.4	960	33.0	856	33.2	799	30.6	788	32.0	732	30.2	726	31.7
Missing	3	0.1	1	0.1	0	0	2	0.1	5	0.2	2	0.1	0	0	1	0.0
Child-level characteristics																
Sex																
Male	1076	50.3	908	49.7	1507	51.8	1348	52.3	1334	51.1	1251	50.8	1240	51.1	1177	51.4
Female	1062	49.7	918	50.3	1401	48.2	1231	47.7	1278	48.9	1211	49.2	1187	48.9	1113	48.6
Age (in months)																
Median/IQR	31	17,44	28	16,42	29	15,44	27	15,43	28	15,40	27	15,41	27	15,41	28	15,41
0-11	302	14.1	280	15.3	496	17.1	441	17.1	451	17.3	423	17.2	413	17.0	373	16.3
12-23	474	22.2	442	24.2	693	23.8	633	24.5	626	24.0	645	26.2	623	25.7	555	24.2
24-35	463	21.7	419	23.0	572	19.7	523	20.3	622	23.8	540	21.9	541	22.3	530	23.1
36-59	899	42.1	685	37.5	1147	39.4	982	38.1	913	35.0	854	34.7	850	35.0	832	36.3

Notes: *Women's mobility is coded categorically based on them reporting having ever been to the marketplace, health centre, women's group, or outside the village (never been to any, been to some or all but never alone, been to at least one alone). HHs=households.

Table S4: Baseline cluster-level characteristics by arm among entire clusters lost due to violent insecurity during the follow-up period.

	Intervention N=3		Control N=3	
	N	%	N	%
Population size (people)				
Median/IQR	374	148, 443	489	309, 513
<700	3	100	3	100
=>700	0	0	0	0
Distance from PHC (in km)				
Median/IQR	8.2	7.3, 12.5	6.4	4.8, 6.8
<=5.0	0	0	1	33.3
>5.0	3	100	2	66.7
Topography				
None	2	66.7	3	100
On clifftop	1	33.3	0	0
PHC inaccessible during rainy season (June, July, August)	0	0	0	0
CHW services available				
None	2	66.7	1	33.3
Satellite village	1	33.3	2	66.7
Posted village	0	0	0	0
PHC catchment area				
Dimbal	1	33.3	0	0
Lessagou	0	0	0	0
Doundé	0	0	0	0
Ende	0	0	0	0
Soubala	0	0	0	0
Kanibozon	2	66.7	1	33.3
Koulongon	0	0	2	66.7

Table S5: Sample characteristics (A) between sick under-five child-year observations with complete and missing data on any general health-care utilisation outcome throughout the three-year follow-up period, and (B) between arms among those child-years with missing outcome data.

	A				B			
	Complete N=18,743 child-years		Missing N=1362 child-years		Intervention N=744 child-years		Control N=618 child-years	
	N	%	N	%				
Catchment area								
Dimbal	4237	22.6	313	23.0	132	17.7	181	29.3
Lessagou	3023	16.1	191	14.0	141	19.0	50	8.1
Doundé	2349	12.5	140	10.3	89	12.0	51	8.3
Ende	883	4.7	67	4.9	39	5.2	28	4.5
Soubala	3729	19.9	305	22.4	159	21.4	146	23.6
Kanibozon	1792	9.6	130	9.5	83	11.2	47	7.6
Koulongon	2730	14.6	216	15.9	101	13.6	115	18.6
Household-level characteristics								
Household size								
Median/IQR	8	5, 11	8	5, 11	8	5, 11	8	5, 11
Household wealth quintile								
Poorest	3340	17.8	226	16.6	141	19.0	85	13.8
Poor	3574	19.1	243	17.8	114	15.3	129	20.9
Middle	3771	20.1	291	21.4	169	22.7	122	19.7
Rich	4014	21.4	285	20.9	136	18.3	149	24.1
Richest	3988	21.3	313	23.0	182	24.5	131	21.2
Missing	56	0.3	4	0.3	2	0.3	2	0.3
Mother-level characteristics								
Marital status								
Not married	185	1.0	12	0.9	9	1.2	3	0.5
Polygynous	8475	45.2	645	47.4	358	48.1	287	46.4
Monogamous	10078	53.8	704	51.7	377	50.7	327	52.9
Missing	5	0.0	1	0.1	0	0	1	0.2
Parity (reported live births)								
Median/IQR	4	3, 7	5	3, 7	5	3, 7	5	3, 7
1-2	4313	23.0	295	21.7	166	22.3	129	20.9
3-4	5143	27.4	366	26.9	205	27.6	161	26.1

5-6	4382	23.4	329	24.2	160	21.5	169	27.4
7+	4905	26.2	372	27.3	213	28.6	159	25.7
Mother's education								
None	15836	84.5	1153	84.7	620	83.3	533	86.3
Any	1723	9.2	132	9.7	69	9.3	63	10.2
Missing	1184	6.3	77	5.7	55	7.4	22	3.6
Mother participates in paid labour								
No	15396	82.1	1137	83.5	628	84.4	509	82.4
Yes	3317	17.7	224	16.5	115	15.5	109	17.6
Missing	30	0.2	1	0.1	1	0.1	0	0
Mother's mobility								
No place	5330	28.4	376	27.6	191	25.7	185	29.9
Any place but none alone	3164	16.9	211	15.5	114	15.3	97	15.7
Any place alone	8885	47.4	680	49.9	378	50.8	302	48.9
Missing	1364	7.3	95	7.0	61	8.2	34	5.5
Mother's decision-making								
None	12871	68.7	933	68.5	506	68.0	427	69.1
Any	5857	31.3	427	31.4	237	31.9	190	30.7
Missing	15	0.1	2	0.2	1	0.1	1	0.2
Child-level characteristics								
Sex								
Male	9618	51.3	710	52.1	381	51.2	329	53.2
Female	9125	48.7	652	47.9	363	48.8	289	46.8
Age (in months)								
Median/IQR	27	14, 42	27	15, 41	27	15, 40	27	15, 42
0-11	3697	19.7	228	16.7	125	16.8	103	16.7
12-23	4397	23.5	357	26.2	196	26.3	161	26.1
24-35	3944	21.0	310	22.8	169	22.7	141	22.8
36-59	6705	35.8	467	34.3	254	34.1	213	34.5

Table S6: Sample characteristics (A) between sick with iCCM illness child-year observations with complete and missing data on any recommended case management outcome throughout the three-year follow-up period, and (B) between arms among those child-years with missing outcome data.

	A				B			
	Complete<br>N=14,661 child-years		Missing<br>N=617 child-years		Intervention<br>N=336 child-years		Control<br>N=281 child-years	
	N	%	N	%				
Catchment area								
Dimbal	3314	22.6	137	22.2	56	16.7	81	28.8
Lessagou	2414	16.5	86	13.9	59	17.6	27	9.6
Doundé	1776	12.1	65	10.5	33	9.8	32	11.4
Ende	651	4.4	34	5.5	26	7.7	8	2.9
Soubala	2917	19.9	152	24.6	85	25.3	67	23.8
Kanibozon	1359	9.3	51	8.3	33	9.8	18	6.4
Koulongon	2230	15.2	92	14.9	44	13.1	48	17.1
Household-level characteristics								
Household size								
Median/IQR	8	5, 11	8	5, 11	8	5, 11	7	5, 11
Household wealth quintile								
Poorest	2574	17.6	105	17.0	64	19.1	41	14.6
Poor	2760	18.8	110	17.8	56	16.7	54	19.2
Middle	2993	20.4	119	19.3	65	19.4	54	19.2
Rich	3112	21.2	133	21.6	64	19.1	69	24.6
Richest	3183	21.7	146	23.7	85	25.3	61	21.7
Missing	39	0.3	4	0.7	2	0.6	2	0.7
Mother-level characteristics								
Marital status								
Not married	137	0.9	6	1.0	6	1.8		
Polygynous	6711	45.8	301	48.8	179	53.3	122	43.4
Monogamous	7810	53.3	309	50.1	151	44.9	158	56.2
Missing	3	0.0	1	0.2	0	0	1	0.4
Parity (reported live births)								
Median/IQR	5	3, 7	4	3, 7	5	3, 7	4	3, 6
1-2	3303	22.5	148	24.0	79	23.5	69	24.6
3-4	4007	27.3	166	26.9	83	24.7	83	29.5

5-6	3435	23.4	134	21.7	69	20.5	65	23.1
7+	3916	26.7	169	27.4	105	31.3	64	22.8
Mother's education								
None	12429	84.8	528	85.6	283	84.2	245	87.2
Any	1320	9.0	54	8.8	27	8.0	27	9.6
Missing	912	6.2	35	5.7	26	7.7	9	3.2
Mother participates in paid labour								
No	12081	82.4	516	83.6	286	85.1	230	81.9
Yes	2557	17.4	101	16.4	50	14.9	51	18.2
Missing	23	0.2	0	0	0	0	0	0
Mother's mobility								
No place	4123	28.1	170	27.6	89	26.5	81	28.8
Any place but none alone	2460	16.8	99	16.1	53	15.8	46	16.4
Any place alone	7022	47.9	305	49.4	165	49.1	140	49.8
Missing	1056	7.2	43	7.0	29	8.6	14	5.0
Mother's decision-making								
None	9999	68.2	408	66.1	216	64.3	192	68.3
Any	4653	31.7	208	33.7	119	35.4	89	31.7
Missing	9	0.1	1	0.2	1	0.3	0	0
Child-level characteristics								
Sex								
Male	7533	51.4	324	52.5	173	51.5	151	53.7
Female	7128	48.6	293	47.5	163	48.5	130	46.3
Age (in months)								
Median/IQR	28	15, 42	24	14, 37	26	15, 38	23	13, 35
0-11	2490	17.0	107	17.3	50	14.9	57	20.3
12-23	3588	24.5	187	30.3	102	30.4	85	30.3
24-35	3174	21.7	154	25.0	85	25.3	69	24.6
36-59	5409	36.9	169	27.4	99	29.5	70	24.9

Table S7: Sample characteristics (A) between child-year observations with complete and missing data on any disease prevalence outcome throughout the three-year follow-up period, and (B) between arms among those child-years with missing outcome data.

	A				B			
	Complete<br>N=46,691 child-years		Missing<br>N=98 child-years		Intervention<br>N=48 child-years		Control<br>N=50 child-years	
	N	%	N	%				
Catchment area								
Dimbal	10419	22.3	22	22.5	9	18.8	13	26.0
Lessagou	6950	14.9	15	15.3	9	18.8	6	12.0
Doundé	6037	12.9	12	12.2	7	14.6	5	10.0
Ende	2464	5.3	6	6.1	5	10.4	1	2.0
Soubala	9206	19.7	14	14.3	8	16.7	6	12.0
Kanibozon	4760	10.2	9	9.2	6	12.5	3	6.0
Koulongon	6855	14.7	20	20.4	4	8.3	16	32.0
Household-level characteristics								
Household size								
Median/IQR	8	5, 11	7	5, 10	8	6, 10.5	7	5, 10
Household wealth quintile								
Poorest	8321	17.8	21	21.4	9	18.8	12	24.0
Poor	8975	19.2	28	28.6	12	25.0	16	32.0
Middle	9315	20.0	23	23.5	10	20.8	13	26.0
Rich	9806	21.0	17	17.4	11	22.9	6	12.0
Richest	10124	21.7	9	9.2	6	12.5	3	6.0
Missing	150	0.3	0	0	0	0	0	0
Mother-level characteristics								
Marital status								
Not married	444	1.0	2	2.0	1	2.1	1	2.0
Polygynous	21181	45.4	47	48.0	28	58.3	19	38.0
Monogamous	25054	53.7	48	49.0	19	39.6	29	58.0
Missing	12	0.0	1	1.0	0	0	1	2.0
Parity (reported live births)								
Median/IQR	4	3, 7	4	3, 7	3.5	2, 6.5	4	3, 7
1-2	10281	22.0	24	24.5	13	27.1	11	22.0
3-4	13421	28.7	32	32.7	15	31.3	17	34.0
5-6	11162	23.9	17	17.4	8	16.7	9	18.0

7+	11827	25.3	25	25.5	12	25.0	13	26.0
Mother's education								
None	39259	84.1	82	83.7	40	83.3	42	84.0
Any	4408	9.4	9	9.2	4	8.3	5	10.0
Missing	3024	6.5	7	7.1	4	8.3	3	6.0
Mother participates in paid labour								
No	39117	83.8	87	88.8	41	85.4	46	92.0
Yes	7506	16.1	11	11.2	7	14.6	4	8.0
Missing	68	0.2	0	0	0	0	0	0
Mother's mobility								
No place	13569	29.1	32	32.7	19	39.6	13	26.0
Any place but none alone	8033	17.2	9	9.2	3	6.3	6	12.0
Any place alone	21621	46.3	47	48.0	19	39.6	28	56.0
Missing	3468	7.4	10	10.2	7	14.6	3	6.0
Mother's decision-making								
None	32748	70.1	56	57.1	27	56.3	29	58.0
Any	13900	29.8	42	42.9	21	43.8	21	42.0
Missing	43	0.1	0	0	0	0	0	0
Child-level characteristics								
Sex								
Male	23580	50.5	55	56.1	28	58.3	27	54.0
Female	23111	49.5	43	43.9	20	41.7	23	46.0
Age (in months)								
Median/IQR	37	22, 49	28	14, 43	37	24, 43.5	36.5	18, 51
0-11	9200	19.7	15	15.3	6	12.5	9	18.0
12-23	10098	21.6	13	13.3	6	12.5	7	14.0
24-35	9558	20.5	16	16.3	8	16.7	8	16.0
36-59	17835	38.2	54	55.1	28	58.3	26	52.0

Table S8: Sensitivity analyses on the primary outcome, any prompt treatment, recommended case management, and prompt, recommended case management due to missing data, especially at 24 months

	Model 1: Assume experienced event			Model 2: Assume did not experience event			Model 3: MICE		
	AOR_{CS} (C vs I)	95% CIs	p-value	AOR_{CS} (C vs I)	95% CIs	p-value	AOR_{CS} (C vs I)	95% CIs	p-value
Prompt treatment within health sector									
Overall	1.12	1.00, 1.26	0.059	1.08	0.96, 1.20	0.189	1.12	0.99, 1.26	0.066
Time-point									
12-months	1.22	1.06, 1.41	0.006	1.22	1.07, 1.40	0.003	1.23	1.07, 1.42	0.004
24-months	1.06	0.92, 1.22	0.438	0.93	0.81, 1.07	0.333	1.03	0.89, 1.19	0.690
36-months	1.08	0.94, 1.25	0.278	1.07	0.94, 1.23	0.308	1.09	0.95, 1.26	0.235
LRT			0.0954			0.0009			0.0136, 0.0908
Any prompt treatment									
Overall	1.14	1.01, 1.27	0.031	1.08	0.98, 1.19	0.143	1.12	1.00, 1.26	0.047
Time-point									
12-months	1.24	1.07, 1.42	0.003	1.23	1.08, 1.40	0.002	1.24	1.08, 1.43	0.002
24-months	1.04	0.91, 1.20	0.559	0.92	0.81, 1.04	0.194	1.00	0.87, 1.16	0.965
36-months	1.13	0.98, 1.30	0.094	1.11	0.98, 1.27	0.114	1.13	0.98, 1.30	0.086
LRT			0.0651			0.0002			0.0038, 0.1896
Recommended case management									
Overall	1.09	0.97, 1.21	0.153	1.07	0.96, 1.20	0.216	1.08	0.97, 1.21	0.165
Time-point									
12-months	1.05	0.90, 1.21	0.541	1.07	0.92, 1.25	0.368	1.06	0.91, 1.24	0.431
24-months	1.18	1.02, 1.38	0.029	1.13	0.96, 1.32	0.139	1.16	0.99, 1.36	0.066
36-months	1.03	0.88, 1.21	0.729	1.02	0.86, 1.20	0.818	1.03	0.87, 1.21	0.762
LRT			0.2395			0.6072			0.3559, 0.7118
Prompt, recommended case management									
Overall	1.10	0.98, 1.23	0.110	1.08	0.97, 1.22	0.170	1.09	0.97, 1.23	0.139
Time-point									
12-months	1.06	0.91, 1.23	0.464	1.08	0.93, 1.27	0.300	1.08	0.93, 1.26	0.334
24-months	1.24	1.06, 1.44	0.006	1.19	1.01, 1.40	0.033	1.21	1.03, 1.43	0.019
36-months	1.00	0.85, 1.17	0.981	0.98	0.83, 1.15	0.776	0.98	0.83, 1.16	0.849
LRT			0.0448			0.1393			0.2162, 0.3454

Notes: We assumed all child-year observations with missing outcome data experienced the event in model 1 and assumed they all did not in model 2. We performed multiple imputation by chained equations (MICE) in model 3. Following MICE, we report a joint test of coefficients between treatment arm and 24 months, and treatment arm and 36 months, in place of the likelihood ratio test (LRT).

Table S9: Heterogeneous treatment effects by cluster population size, cluster distance to nearest PHC, and household wealth on the secondary health-care utilisation outcomes at each time-point

	12 months					24 months*					36 months				
	ARC	ARI	AOR_{CS}	95% CIs	p-value	ARC	ARI	AOR_{CS}	95% CIs	p-value	ARC	ARI	AOR_{CS}	95% CIs	p-value
Prompt treatment within the health sector															
Cluster distance to PHC (km)															
<=5.0	0.61	0.64	1.12	0.87, 1.45	0.375	··	··	0.97	0.71, 1.31	0.833	0.52	0.53	1.05	0.83, 1.32	0.701
>5.0	0.57	0.61	1.18	0.96, 1.45	0.107	··	··	1.22	0.94, 1.57	0.131	0.51	0.54	1.13	0.93, 1.38	0.208
LRT					0.7533					0.2639					0.6120
Cluster population size (people)															
<700	0.63	0.67	1.20	0.94, 1.53	0.152	··	··	1.21	0.89, 1.63	0.223	0.51	0.56	1.24	0.97, 1.59	0.081
=>700	0.56	0.60	1.18	0.98, 1.42	0.087	··	··	1.03	0.80, 1.33	0.819	0.52	0.52	1.03	0.85, 1.24	0.779
LRT					0.9143					0.4393					0.2254
Household wealth															
Less poor	0.60	0.63	1.14	0.97, 1.34	0.122	··	··	1.04	0.86, 1.27	0.663	0.52	0.54	1.07	0.91, 1.25	0.397
Poorest	0.55	0.60	1.21	0.92, 1.58	0.169	··	··	1.41	1.04, 1.91	0.028	0.49	0.54	1.22	0.94, 1.57	0.131
LRT					0.6599					0.0370					0.3300
Any prompt treatment*															
Cluster distance to PHC (km)															
<=5.0	0.68	0.70	1.08	0.84, 1.38	0.551	··	··	1.09	0.80, 1.47	0.588	0.58	0.59	1.06	0.84, 1.34	0.619
>5.0	0.63	0.67	1.21	0.99, 1.47	0.059	··	··	1.08	0.85, 1.38	0.538	0.55	0.59	1.16	0.96, 1.41	0.121
LRT					0.4810					0.9751					0.5435
Cluster population size (people)															
<700	0.67	0.71	1.22	0.95, 1.56	0.115	··	··	1.30	1.00, 1.75	0.083	0.56	0.61	1.23	0.96, 1.58	0.094
=>700	0.64	0.67	1.14	0.95, 1.37	0.170	··	··	0.95	0.75, 1.21	0.677	0.57	0.58	1.06	0.88, 1.28	0.533
LRT					0.6550					0.1039					0.3380
Household wealth															
Less poor	0.66	0.68	1.12	0.95, 1.31	0.181	··	··	1.06	0.87, 1.29	0.557	0.57	0.59	1.08	0.92, 1.26	0.346
Poorest	0.60	0.66	1.30	0.99, 1.70	0.056	··	··	1.17	0.87, 1.59	0.298	0.54	0.61	1.30	1.01, 1.68	0.043
LRT					0.2671					0.4759					0.1536
Health sector evaluation															
Cluster distance to PHC (km)															
<=5.0	0.66	0.67	1.07	0.82, 1.39	0.613	0.68	0.67	0.93	0.68, 1.26	0.637	0.57	0.59	1.05	0.84, 1.33	0.659
>5.0	0.62	0.66	1.17	0.95, 1.45	0.129	0.61	0.66	1.22	0.94, 1.57	0.129	0.57	0.61	1.18	0.97, 1.43	0.100
LRT					0.5819					0.1846					0.4748
Cluster population size (people)															
<700	0.67	0.72	1.27	0.98, 1.63	0.065	0.67	0.68	1.07	0.79, 1.45	0.668	0.57	0.64	1.36	1.07, 1.73	0.013
=>700	0.62	0.64	1.09	0.90, 1.32	0.357	0.62	0.65	1.11	0.86, 1.44	0.403	0.57	0.58	1.03	0.86, 1.23	0.770

LRT					0.3613					0.8374					0.0725
Household wealth															
Less poor	0.64	0.67	1.11	0.94, 1.31	0.239	0.65	0.66	1.02	0.83, 1.25	0.847	0.57	0.60	1.13	0.97, 1.33	0.128
Poorest	0.61	0.65	1.20	0.91, 1.58	0.196	0.59	0.68	1.47	1.09, 1.99	0.012	0.58	0.60	1.12	0.87, 1.44	0.390
LRT					0.5600					0.0098					0.9338
Any care															
Cluster distance to PHC (km)															
<=5.0	0.73	0.73	1.03	0.79, 1.33	0.844	0.74	0.75	1.05	0.79, 1.38	0.743	0.65	0.67	1.10	0.88, 1.37	0.395
>5.0	0.69	0.73	1.20	0.98, 1.48	0.076	0.72	0.75	1.17	0.92, 1.47	0.195	0.61	0.66	1.21	1.01, 1.45	0.040
LRT					0.3460					0.5663					0.5105
Cluster population size (people)															
<700	0.72	0.77	1.35	1.04, 1.75	0.024	0.73	0.77	1.22	0.92, 1.63	0.173	0.63	0.70	1.38	1.09, 1.74	0.007
=>700	0.70	0.71	1.05	0.86, 1.27	0.638	0.73	0.74	1.06	0.85, 1.33	0.592	0.63	0.65	1.08	0.92, 1.27	0.358
LRT					0.1258					0.4526					0.0945
Household wealth															
Less poor	0.71	0.73	1.08	0.91, 1.38	0.386	0.73	0.75	1.08	0.90, 1.29	0.435	0.63	0.66	1.16	1.00, 1.34	0.052
Poorest	0.66	0.72	1.34	1.01, 1.78	0.043	0.71	0.76	1.32	0.97, 1.78	0.075	0.63	0.67	1.20	0.93, 1.54	0.165
LRT					0.1299					0.1850					0.8047
Recommended case management*															
Cluster distance to PHC (km)															
<=5.0	0.48	0.46	0.93	0.72, 1.19	0.552	··	··	1.22	0.88, 1.69	0.226	0.35	0.35	0.97	0.78, 1.21	0.782
>5.0	0.44	0.46	1.15	0.94, 1.40	0.171	··	··	1.26	0.95, 1.66	0.105	0.34	0.35	1.06	0.88, 1.28	0.522
LRT					0.1870					0.8927					0.5315
Cluster population size (people)															
<700	0.47	0.50	1.20	0.91, 1.59	0.187	··	··	1.57	1.11, 2.23	0.012	0.38	0.37	0.96	0.73, 1.26	0.758
=>700	0.45	0.45	1.01	0.85, 1.21	0.882	··	··	1.09	0.84, 1.41	0.537	0.33	0.34	1.06	0.90, 1.25	0.473
LRT					0.3053					0.0951					0.5247
Household wealth															
Less poor	0.46	0.46	1.04	0.88, 1.22	0.687	··	··	1.18	0.94, 1.47	0.147	0.34	0.35	1.04	0.89, 1.22	0.592
Poorest	0.44	0.45	1.08	0.77, 1.50	0.661	··	··	1.57	1.07, 2.30	0.021	0.36	0.35	0.94	0.69, 1.28	0.677
LRT					0.8238					0.1372					0.5335
Prompt, recommended case management*															
Cluster distance to PHC (km)															
<=5.0	0.46	0.44	0.91	0.71, 1.17	0.479	··	··	1.29	0.93, 1.80	0.124	0.31	0.30	0.97	0.77, 1.22	0.797
>5.0	0.40	0.43	1.17	0.96, 1.43	0.125	··	··	1.35	1.01, 1.79	0.040	0.30	0.30	0.99	0.82, 1.20	0.930
LRT					0.1333					0.8550					0.8884
Cluster population size (people)															
<700	0.44	0.46	1.09	0.83, 1.44	0.539	··	··	1.74	1.22, 2.49	0.002	0.34	0.32	0.90	0.69, 1.19	0.477

=>700	0.41	0.42	1.07	0.89, 1.29	0.480	··	··	1.12	0.86, 1.47	0.396	0.29	0.29	1.03	0.87, 1.22	0.740
LRT					0.9053					0.0573					0.4386
Household wealth															
Less poor	0.43	0.43	1.03	0.87, 1.23	0.703	··	··	1.24	0.99, 1.56	0.062	0.30	0.30	0.96	0.82, 1.13	0.623
Poorest	0.40	0.42	1.12	0.80, 1.56	0.500	··	··	1.72	1.18, 2.52	0.005	0.30	0.31	1.06	0.77, 1.45	0.730
LRT					0.6491					0.0871					0.5908

Notes: For every outcome, we ran three separate models, one for each of the predefined effect modifiers that included an interaction term between treatment arm and the modifier, on each of the three annual subsets. We treated each follow-up child sample separately to avoid the complexity that would have been introduced by a triple interaction term between arm, modifier, and time-point. All models controlled for the same covariates as the main model (table 3); we removed the baseline cluster-level summary of wealth in the models that assessed heterogeneous effects by this variable at the household level. We report the results of the likelihood ratio tests (LRT) for interaction between arm and modifier in each model.
[*]On the 24-month subset, we performed multiple imputation by chained equations prior to assessing heterogeneous treatment effects on the following outcomes: any prompt treatment, recommended case management, and prompt, recommended case management. In these cases, we were unable to estimate absolute risks in each arm using the margins command and we used the mi test postestimation command to perform a joint test of coefficients in place of the LRT.

Table S10: Heterogeneous treatment effects by cluster population size, cluster distance to nearest PHC, and household wealth for primary and secondary health-care utilisation outcomes during the three-year trial period overall

Outcome	ARC	ART	OR_{CS}	95% CIs	p-value	Outcome	ARC	ART	OR_{CS}	95% CIs	p-value
Prompt treatment within the health sector						Prompt treatment					
Cluster distance to PHC						Cluster distance to PHC					
<=5.0 km	0.54	0.55	1.01	0.84, 1.22	0.918	<=5.0 km	0.61	0.62	1.05	0.88, 1.26	0.577
>5.0 km	0.50	0.54	1.18	1.01, 1.38	0.039	>5.0 km	0.57	0.61	1.17	1.01, 1.36	0.037
LRT					0.2193	LRT					0.3723
Cluster population size						Cluster population size					
<700 people	0.53	0.57	1.18	0.99, 1.41	0.072	<700 people	0.59	0.63	1.23	1.03, 1.46	0.021
=>700	0.51	0.53	1.07	0.91, 1.25	0.419	=>700	0.59	0.60	1.05	0.91, 1.22	0.520
LRT					0.4132	LRT					0.1826
Household wealth						Household wealth					
Less poor	0.53	0.55	1.08	0.95, 1.22	0.243	Less poor	0.59	0.61	1.09	0.97, 1.23	0.154
Poorest quintile	0.49	0.54	1.23	1.03, 1.46	0.022	Poorest quintile	0.56	0.61	1.25	1.05, 1.48	0.012
LRT					0.1000	LRT					0.0917
Care within the health sector						Any care					
Cluster distance to PHC						Cluster distance to PHC					
<=5.0 km	0.64	0.64	1.01	0.84, 1.23	0.886	<=5.0 km	0.71	0.72	1.07	0.89, 1.28	0.480
>5.0 km	0.60	0.64	1.19	1.02, 1.40	0.026	>5.0 km	0.67	0.71	1.21	1.04, 1.40	0.011
LRT					0.1940	LRT					0.2928
Cluster population size						Cluster population size					
<700 people	0.63	0.68	1.21	1.02, 1.44	0.032	<700 people	0.69	0.74	1.31	0.11, 1.56	0.002
=>700	0.61	0.62	1.07	0.92, 1.24	0.398	=>700	0.69	0.70	1.06	0.92, 1.22	0.436
LRT					0.2868	LRT					0.0589
Household wealth						Household wealth					
Less poor	0.63	0.64	1.09	0.96, 1.23	0.179	Less poor	0.69	0.71	1.12	1.00, 1.26	0.060
Poorest quintile	0.60	0.64	1.24	1.04, 1.47	0.015	Poorest quintile	0.67	0.72	1.28	1.07, 1.52	0.006
LRT					0.0959	LRT					0.1061
Prompt, recommended case management						Recommended case management					
Cluster distance to PHC						Cluster distance to PHC					
<=5.0 km	0.38	0.39	1.03	0.86, 1.24	0.725	<=5.0 km	0.43	0.43	1.02	0.85, 1.22	0.834
>5.0 km	0.34	0.37	1.14	0.98, 1.32	0.095	>5.0 km	0.40	0.42	1.12	0.97, 1.20	0.120
LRT					0.4277	LRT					0.4078
Cluster population size						Cluster population size					
<700 people	0.37	0.40	1.16	0.96, 1.40	0.114	<700 people	0.42	0.45	1.17	0.97, 1.41	0.092
=>700	0.35	0.36	1.06	0.92, 1.22	0.439	=>700	0.40	0.41	1.04	0.90, 1.19	0.615

LRT					0.4290	LRT					0.2954
Household wealth						Household wealth					
Less poor	0.36	0.37	1.05	0.93, 1.19	0.400	Less poor	0.41	0.42	1.06	0.94, 1.20	0.310
Poorest quintile	0.35	0.38	1.23	1.00, 1.51	0.050	Poorest quintile	0.40	0.42	1.12	0.91, 1.38	0.267
LRT					0.1446	LRT					0.6117

Table S11: Characteristics of sick child-year observations that met the per-protocol threshold and those that did not in each arm.

	Intervention				Control			
	Per-protocol N=4864 child-years		Not per-protocol N=5478 child-years		Per-protocol N=7561 child-years		Not per-protocol N=2098 child-years	
	N	%	N	%				
Catchment area								
Dimbal	776	16.0	984	18.0	2294	30.3	491	23.4
Lessagou	853	17.5	1183	21.6	862	11.4	287	13.7
Doundé	784	16.1	730	13.3	736	9.7	234	11.2
Ende	276	5.7	222	4.1	344	4.6	80	3.8
Soubala	938	19.3	938	17.1	1688	22.3	467	22.3
Kanibozon	639	13.1	625	11.4	479	6.3	157	7.5
Koulongon	598	12.3	796	14.5	1158	15.3	382	18.2
Household-level characteristics								
Distance to PHC								
Median/IQR (km)	6.1	3.9, 7.6	6.1	4.0, 7.7	5.4	2.7, 8.0	5.5	3.1, 7.8
Household size								
Median/IQR	8	6, 12	8	5, 11	8	5, 11	9	6, 12
Household wealth quintile								
Poorest	859	17.7	1038	19.0	1303	17.2	337	16.1
Poor	874	18.0	994	18.2	1536	20.3	398	19.0
Middle	950	19.5	1169	21.3	1508	19.9	424	20.2
Rich	1009	20.7	1151	21.0	1647	21.8	468	22.3
Richest	1154	23.7	1105	20.2	1549	20.5	468	22.3
Missing	18	0.4	21	0.4	18	0.2	3	0.1
Mother-level characteristics								
Mother's marital status								
Not married	49	1.0	53	1.0	72	1.0	15	0.7
Polygynous	2521	51.8	2217	40.5	3243	42.9	1109	52.9
Monogamous	2291	47.1	3208	58.6	4244	56.1	973	46.4
Missing	3	0.1	53	1.0	2	0.0	1	0.1
Mother's parity (# live births)								
Median/IQR	5	3, 7	4	3, 7	4	3, 7	5	3, 7
1-2	1017	20.9	1341	24.5	1771	23.4	446	21.3

3-4	1317	27.1	1479	27.0	2124	28.1	567	27.0
5-6	1204	24.8	1247	22.8	1727	22.8	516	24.6
7+	1326	27.3	1411	25.8	1939	25.6	569	27.1
Mother's education								
None	4140	85.1	4583	83.7	6395	84.6	1790	85.3
Any	404	8.3	492	9.0	750	9.9	190	9.1
Missing	320	6.6	403	7.4	416	5.5	118	5.6
Mother participates in paid labour								
No	3921	80.6	4533	82.8	6323	83.6	1680	80.1
Yes	936	19.2	940	17.2	1230	16.3	407	19.4
Missing	7	0.1	5	0.1	8	0.1	11	0.5
Mother's mobility								
No place	1420	29.2	1449	26.5	2231	29.5	592	28.2
Any place but none alone	743	15.3	945	17.3	1309	17.3	357	17.0
Any place alone	2343	48.2	2613	47.7	3533	46.7	1012	48.2
Missing	358	7.4	471	8.6	488	6.5	137	6.5
Mother's decision-making								
None	3452	71.0	3687	67.3	5036	66.6	1570	74.8
Any	1409	29.0	1786	32.6	2519	33.3	525	25.0
Missing	3	0.1	5	0.1	6	0.1	3	0.1
Child-level characteristics								
Sex								
Male	2556	52.6	2733	49.9	3911	51.7	1074	51.2
Female	2308	47.5	2745	50.1	3650	48.3	1024	48.8
Age (in months)								
Median/IQR	28	14, 42	27	14, 41	27	14, 41	27	14, 42
0-11	952	19.6	1072	19.6	1476	19.5	402	19.2
12-23	1095	22.5	1329	24.3	1796	23.8	506	24.1
24-35	1046	21.5	1138	20.8	1633	21.6	423	20.2
36-59	1771	36.4	1939	35.4	2656	35.1	767	36.6

Table S12: Per-protocol subgroup estimates for the primary and secondary health-care utilisation outcomes, excluding observations in the intervention arm that did not receive at least two CHW home visits as well as observations in the control arm that received any CHW home visit in the month preceding the survey, during the three-year trial period overall[1] and at each follow-up time-point[2]

Outcome	ARC	ARI	AOR_{CS} (C vs I)	95% CIs	p-value
Prompt treatment within the health sector (N=11,529)					
Overall[1]	0.52	0.57	1.22	1.06, 1.41	0.006
Time-point[2]					
12 months	0.58	0.66	1.44	1.21, 1.72	<0.001
24 months	0.46	0.47	1.08	0.90, 1.30	0.414
36 months	0.52	0.55	1.14	0.95, 1.36	0.175
Likelihood ratio test					0.0065
Prompt treatment (N=11,524)					
Overall[1]	0.59	0.64	1.25	1.09, 1.44	0.002
Time-point[2]					
12 months	0.64	0.71	1.42	1.19, 1.70	<0.001
24 months	0.55	0.58	1.09	0.91, 1.32	0.349
36 months	0.56	0.61	1.24	1.03, 1.49	0.021
Likelihood ratio test					0.0336
Health sector evaluation (N=12,416)					
Overall[1]	0.62	0.68	1.28	1.11, 1.48	0.001
Time-point[2]					
12 months	0.63	0.70	1.38	1.16, 1.66	<0.001
24 months	0.65	0.71	1.30	1.09, 1.57	0.004
36 months	0.58	0.61	1.15	0.95, 1.38	0.147
Likelihood ratio test					0.1558
Any care (N=9039)					
Overall[1]	0.69	0.75	1.33	1.15, 1.53	<0.001
Time-point[2]					
12 months	0.71	0.76	1.34	1.12, 1.61	0.002
24 months	0.74	0.79	1.34	1.11, 1.61	0.003
36 months	0.63	0.69	1.30	1.08, 1.56	0.006
Likelihood ratio test					0.9349
Recommended case management (N=9039)					
Overall[1]	0.41	0.45	0.20	0.06, 0.34	0.006
Time-point[2]					
12 months	0.46	0.49	1.21	0.99, 1.47	0.062
24 months	0.39	0.45	1.39	1.14, 1.70	0.001
36 months	0.38	0.39	1.06	0.86, 1.32	0.583
Likelihood ratio test					0.1236
Prompt, recommended case management (N=9039)					
Overall[1]	0.36	0.39	1.19	1.03, 1.37	0.018
Time-point[2]					
12 months	0.42	0.46	1.22	1.01, 1.48	0.043
24 months	0.31	0.36	1.31	1.07, 1.60	0.008
36 months	0.33	0.33	1.02	0.82, 1.26	0.888
Likelihood ratio test					0.1437

Figure S3: Box plot representing the variability in cluster summaries of the prevalence outcomes in the intervention and control arms at each time-point

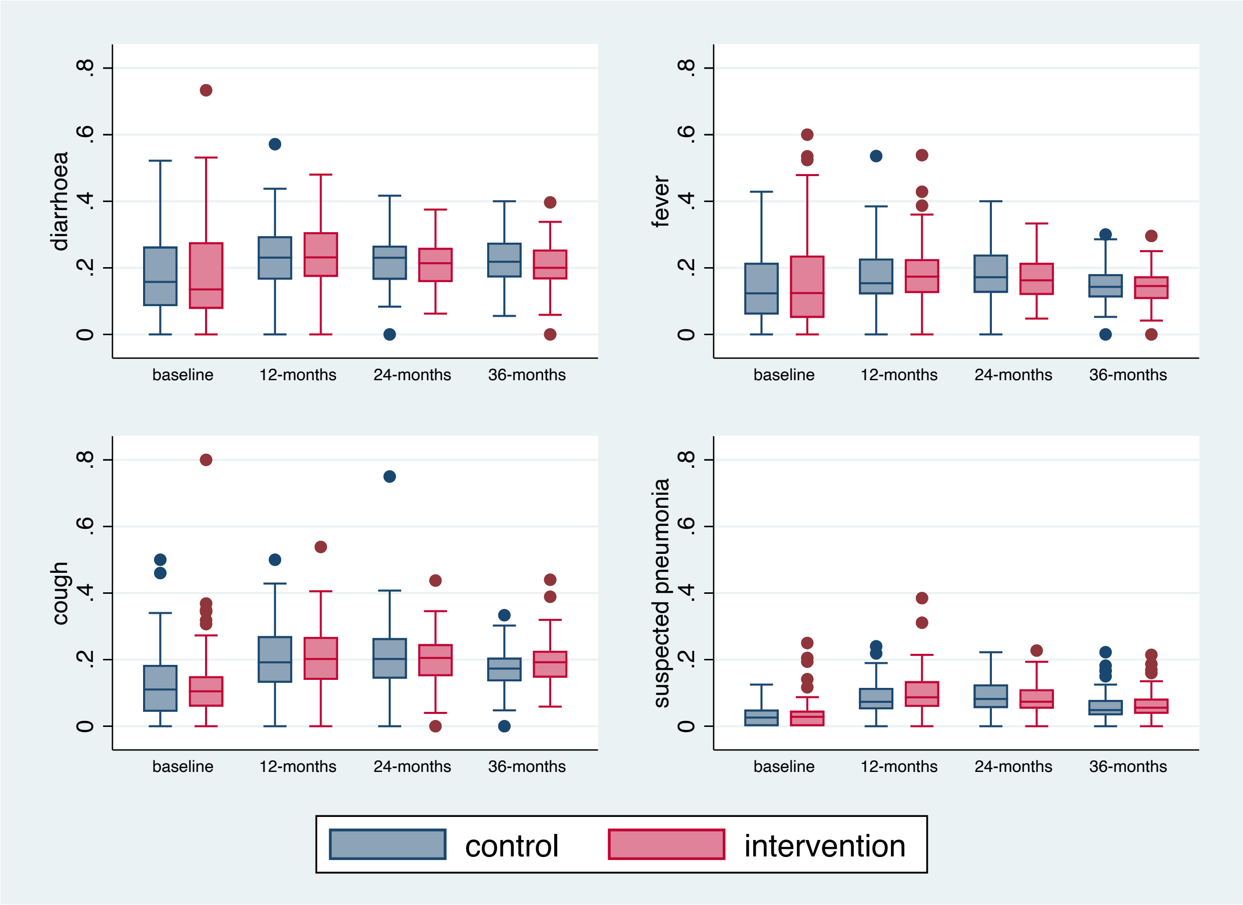

Table S13: Cluster-specific intervention effects on prevalence outcomes, including absolute risks in each arm, during the three-year trial period overall[1] and at each follow-up time-point[2]

Outcome	ARC	ARI	AOR_{CS} (C vs I)	95% CIs	p-value
Fever (N=46,755)					
Overall[1]	0.17	0.17	0.98	0.89, 1.07	0.614
Time-point[2]					
12 months	0.18	0.18	1.02	0.91, 1.14	0.770
24 months	0.18	0.17	0.91	0.81, 1.03	0.125
36 months	0.15	0.15	1.00	0.89, 1.13	0.981
Cluster level ICC					
Control			0.011	0.006, 0.020	
Intervention			0.009	0.005, 0.016	
Child-within-cluster level ICC					
Control			0.107	0.075, 0.150	
Intervention			0.051	0.025, 0.102	
Likelihood ratio test					0.1702
Diarrhoea (N=46,758)					
Overall[1]	0.22	0.22	1.00	0.91, 1.09	0.989
Time-point[2]					
12 months	0.24	0.25	1.06	0.95, 1.19	0.273
24 months	0.22	0.21	0.97	0.87, 1.09	0.636
36 months	0.22	0.21	0.95	0.85, 1.07	0.428
ICC (95% CIs)					
Control			0.007	0.003, 0.014	
Intervention			0.010	0.006, 0.017	
Child-within-cluster level ICC					
Control			0.150	0.121, 0.184	
Intervention			0.141	0.113, 0.175	
Likelihood ratio test					0.1409
Cough (N=46,771)					
Overall[1]	0.19	0.20	1.04	0.95, 1.14	0.366
Time-point[2]					
12 months	0.19	0.21	1.16	1.04, 1.30	0.010
24 months	0.20	0.20	1.01	0.90, 1.13	0.914
36 months	0.19	0.18	0.96	0.86, 1.08	0.516
ICC (95% CIs)					
Control			0.015	0.008, 0.026	
Intervention			0.006	0.003, 0.012	
Child-within-cluster level ICC					
Control			0.108	0.077, 0.148	
Intervention			0.107	0.078, 0.144	
Likelihood ratio test					0.0057
Suspected pneumonia (N=46,733)					
Overall[1]	0.08	0.08	1.06	0.96, 1.18	0.244
Time-point[2]					

12 months	0.08	0.10	1.22	1.07, 1.40	0.003
24 months	0.08	0.08	0.98	0.85, 1.13	0.796
36 months	0.06	0.06	0.96	0.82, 1.12	0.603
ICC (95% CIs)					
Control			0.005	0.001, 0.019	
Intervention			0.010	0.004, 0.022	
Child-within-cluster level ICC					
Control			0.051	0.012, 0.192	
Intervention			0.035	0.006, 0.195	
Likelihood ratio test					0.0058

Table S14: Heterogeneous treatment effects by cluster population size, distance to nearest PHC, and household wealth on the prevalence outcomes at each time-point

	12 months					24 months					36 months				
	ARC	ARI	AOR_{CS}	95% CIs	p-value	ARC	ARI	AOR_{CS}	95% CIs	p-value	ARC	ARI	AOR_{CS}	95% CIs	p-value
Fever															
Cluster distance to PHC (km)															
<=5.0	0.17	0.17	0.96	0.77, 1.19	0.696	0.16	0.18	1.14	0.94, 1.39	0.185	0.14	0.14	0.99	0.82, 1.20	0.916
>5.0	0.18	0.19	0.94	0.79, 1.11	0.449	0.19	0.17	0.85	0.72, 1.00	0.052	0.14	0.16	1.11	0.94, 1.29	0.211
LRT					0.8685					0.0252					0.3802
Cluster population size (people)															
<700	0.18	0.18	0.95	0.77, 1.17	0.616	0.18	0.16	0.83	0.67, 1.02	0.082	0.15	0.15	0.97	0.79, 1.19	0.743
=>700	0.18	0.18	0.95	0.80, 1.13	0.553	0.17	0.18	1.04	0.89, 1.23	0.602	0.14	0.15	1.12	0.96, 1.30	0.140
LRT					0.9855					0.0902					0.2613
Household wealth															
Less poor	0.19	0.18	0.96	0.83, 1.10	0.522	0.18	0.17	0.93	0.81, 1.06	0.292	0.14	0.15	1.06	0.93, 1.21	0.351
Poorest	0.17	0.16	0.89	0.70, 1.13	0.334	0.16	0.18	1.14	0.90, 1.43	0.275	0.15	0.15	1.04	0.83, 1.29	0.755
LRT					0.5498					0.0847					0.8184
Diarrhoea															
Cluster distance to PHC (km)															
<=5.0	0.24	0.24	1.03	0.85, 1.24	0.757	0.23	0.22	0.99	0.83, 1.19	0.947	0.22	0.22	0.98	0.82, 1.17	0.819
>5.0	0.24	0.24	0.99	0.85, 1.15	0.896	0.21	0.21	1.02	0.88, 1.19	0.792	0.21	0.21	1.01	0.87, 1.17	0.889
LRT					0.7486					0.8247					0.7910
Cluster population size (people)															
<700	0.23	0.24	1.03	0.86, 1.25	0.738	0.23	0.21	0.86	0.72, 1.04	0.131	0.23	0.20	0.84	0.68, 1.00	0.045
=>700	0.25	0.24	0.99	0.85, 1.15	0.899	0.21	0.22	1.11	0.96, 1.27	0.163	0.20	0.22	1.11	0.97, 1.28	0.135
LRT					0.7331					0.0420					0.0130
Household wealth															
Less poor	0.24	0.24	0.98	0.87, 1.10	0.720	0.22	0.22	1.01	0.90, 1.14	0.843	0.22	0.22	0.99	0.88, 1.12	0.908
Poorest	0.23	0.26	1.14	0.93, 1.39	0.220	0.21	0.21	1.00	0.81, 1.23	0.986	0.20	0.20	1.02	0.84, 1.25	0.838
LRT					0.1441					0.8949					0.7869
Cough															
Cluster distance to PHC (km)															
<=5.0	0.20	0.21	1.05	0.85, 1.30	0.648	0.20	0.21	1.10	0.89, 1.36	0.388	0.18	0.17	0.96	0.79, 1.16	0.659
>5.0	0.20	0.21	1.07	0.89 1.27	0.472	0.21	0.20	0.95	0.80, 1.14	0.598	0.18	0.20	1.11	0.95, 1.30	0.192
LRT					0.9173					0.3163					0.2434
Cluster population size (people)															
<700	0.21	0.21	1.00	0.81, 1.24	0.999	0.22	0.19	0.83	0.67, 1.03	0.093	0.17	0.20	1.23	1.00, 1.50	0.047
=>700	0.19	0.21	1.11	0.93, 1.33	0.233	0.19	0.21	1.15	0.97, 1.36	0.114	0.18	0.18	0.96	0.82, 1.12	0.580

LRT					0.4460					0.0213					0.0566
Household wealth															
Less poor	0.20	0.21	1.05	0.91, 1.20	0.532	0.20	0.21	1.01	0.88, 1.17	0.873	0.18	0.19	1.08	0.94, 1.23	0.270
Poorest	0.20	0.21	1.09	0.87, 1.36	0.465	0.20	0.20	1.02	0.82, 1.28	0.858	0.19	0.18	0.93	0.75, 1.14	0.481
LRT					0.7235					0.9355					0.1624
Suspected pneumonia															
Cluster distance to PHC (km)															
<=5.0	0.09	0.10	1.08	0.84, 1.38	0.554	0.08	0.09	1.15	0.88, 1.49	0.316	0.06	0.06	0.89	0.68, 1.18	0.425
>5.0	0.08	0.09	1.18	0.96, 1.46	0.124	0.08	0.08	0.96	0.77, 1.20	0.707	0.06	0.06	1.15	0.92, 1.44	0.229
LRT					0.5833					0.3134					0.1700
Cluster population size (people)															
<700	0.09	0.10	1.17	0.90, 1.52	0.251	0.09	0.08	0.87	0.66, 1.16	0.353	0.06	0.07	1.10	0.82, 1.47	0.528
=>700	0.08	0.09	1.12	0.92, 1.37	0.264	0.08	0.09	1.13	0.92, 1.40	0.254	0.06	0.06	1.02	0.82, 1.26	0.883
LRT					0.8165					0.1523					0.6767
Household wealth															
Less poor	0.08	0.09	1.12	0.94, 1.33	0.191	0.08	0.09	1.02	0.85, 1.22	0.808	0.06	0.06	1.04	0.86, 1.26	0.658
Poorest	0.09	0.10	1.13	0.85, 1.51	0.404	0.07	0.08	1.10	0.79, 1.51	0.577	0.06	0.06	0.98	0.72, 1.35	0.924
LRT					0.9454					0.6726					0.7299

Table S15: Heterogeneous treatment effects by cluster population size, cluster distance to nearest PHC, and household wealth for prevalence outcomes during the three-year trial period overall, controlling for the linear effect of time

Outcome	ARC	ARI	OR_{CS}	95% CIs	p-value	Outcome	ARC	ARI	OR_{CS}	95% CIs	p-value
Fever						Diarrhoea					
Cluster distance to PHC						Cluster distance to PHC					
<=5.0 km	0.16	0.16	1.01	0.88, 1.17	0.851	<=5.0 km	0.23	0.23	1.01	0.87, 1.16	0.945
>5.0 km	0.18	0.17	0.95	0.84, 1.07	0.373	>5.0 km	0.22	0.22	0.99	0.88, 1.12	0.897
LRT					0.4838	LRT					0.8929
Cluster population size						Cluster population size					
<700 people	0.17	0.16	0.91	0.79, 1.05	0.178	<700 people	0.23	0.22	0.91	0.79, 1.04	0.163
=>700	0.17	0.17	1.03	0.91, 1.16	0.634	=>700	0.22	0.23	1.07	0.95, 1.21	0.243
LRT					0.1825	LRT					0.0709
Household wealth						Household wealth					
Less poor	0.17	0.17	0.97	0.88, 1.06	0.471	Less poor	0.23	0.23	0.99	0.90, 1.08	0.785
Poorest quintile	0.16	0.16	1.00	0.87, 1.16	0.980	Poorest quintile	0.21	0.22	1.04	0.90, 1.20	0.573
LRT					0.6011	LRT					0.4278
Cough						Suspected pneumonia					
Cluster distance to PHC						Cluster distance to PHC					
<=5.0 km	0.19	0.20	1.02	0.88, 1.18	0.820	<=5.0 km	0.08	0.08	1.02	0.87, 1.20	0.830
>5.0 km	0.19	0.20	1.06	0.94, 1.19	0.335	>5.0 km	0.07	0.08	1.10	0.96, 1.25	0.177
LRT					0.6675	LRT					0.4889
Cluster population size						Cluster population size					
<700 people	0.20	0.20	1.00	0.87, 1.15	0.957	<700 people	0.08	0.08	1.01	0.86, 1.19	0.908
=>700	0.19	0.20	1.08	0.96, 1.21	0.224	=>700	0.07	0.08	1.10	0.97, 1.25	0.130
LRT					0.4542	LRT					0.4007
Household wealth						Household wealth					
Less poor	0.19	0.20	1.05	0.95, 1.15	0.326	Less poor	0.08	0.08	1.06	0.95, 1.18	0.279
Poorest quintile	0.19	0.19	1.00	0.87, 1.15	0.977	Poorest quintile	0.08	0.08	1.07	0.89, 1.28	0.498
LRT					0.5022	LRT					0.9728

Table S16: Per-protocol subgroup estimates for the prevalence outcomes, excluding observations in the intervention arm that did not receive at least two CHW home visits in the month preceding the survey, during the three-year trial period overall[1] and at each follow-up time-point[2]

Outcome	ARC	ARI	AOR_{CS} (C vs I)	95% CIs	p-value
Fever (N=33,838)					
Overall[1]	0.17	0.17	1.04	0.93, 1.15	0.551
Time-point[2]					
12 months	0.18	0.19	1.09	0.95, 1.25	0.212
24 months	0.18	0.18	0.96	0.84, 1.10	0.563
36 months	0.15	0.15	1.06	0.91, 1.23	0.470
Likelihood ratio test					0.2233
Diarrhoea (N=33,839)					
Overall[1]	0.23	0.23	1.03	0.94, 1.14	0.491
Time-point[2]					
12 months	0.24	0.26	1.14	1.00, 1.29	0.042
24 months	0.22	0.22	0.99	0.87, 1.12	0.842
36 months	0.22	0.21	0.97	0.84, 1.11	0.623
Likelihood ratio test					0.0558
Cough (N=33,848)					
Overall[1]	0.19	0.20	1.07	0.96, 1.19	0.245
Time-point[2]					
12 months	0.19	0.22	1.23	1.07, 1.41	0.004
24 months	0.20	0.21	1.01	0.88, 1.16	0.849
36 months	0.19	0.18	0.96	0.83, 1.11	0.546
Likelihood ratio test					0.0027
Suspected pneumonia (N=33,822)					
Overall[1]	0.08	0.08	1.10	0.98, 1.23	0.106
Time-point[2]					
12 months	0.08	0.10	1.21	1.03, 1.41	0.017
24 months	0.08	0.09	1.09	0.93, 1.28	0.280
36 months	0.06	0.06	0.94	0.78, 1.14	0.547
Likelihood ratio test					0.0920

Table S17: Per-protocol subgroup estimates for the prevalence outcomes, excluding observations in the intervention arm that did not receive at least two CHW home visits as well as observations in the control arm that received any CHW home visit in the month preceding the survey, during the three-year trial period overall[1] and at each follow-up time-point[2]

Outcome	ARC	ARI	AOR_{CS} (C vs I)	95% CIs	p-value
Fever (N=28,848)					
Overall[1]	0.17	0.17	1.04	0.93, 1.16	0.483
Time-point[2]					
12 months	0.18	0.19	1.12	0.97, 1.29	0.115
24 months	0.18	0.18	0.95	0.83, 1.09	0.482
36 months	0.15	0.15	1.06	0.91, 1.23	0.448
Likelihood ratio test					0.1164
Diarrhoea (N=28,850)					
Overall[1]	0.22	0.23	1.04	0.94, 1.15	0.499
Time-point[2]					
12 months	0.24	0.26	1.16	1.02, 1.32	0.028
24 months	0.22	0.22	0.99	0.87, 1.13	0.893
36 months	0.22	0.21	0.95	0.83, 1.09	0.479
Likelihood ratio test					0.0262
Cough (N=28,859)					
Overall[1]	0.19	0.20	1.08	0.96, 1.21	0.180
Time-point[2]					
12 months	0.19	0.22	1.26	1.09, 1.45	0.002
24 months	0.20	0.21	1.04	0.90, 1.20	0.617
36 months	0.19	0.18	0.95	0.82, 1.10	0.482
Likelihood ratio test					0.0013
Suspected pneumonia (N=28,833)					
Overall[1]	0.07	0.08	1.11	0.98, 1.23	0.103
Time-point[2]					
12 months	0.08	0.10	1.22	1.03, 1.46	0.023
24 months	0.08	0.09	1.10	0.93, 1.31	0.275
36 months	0.06	0.06	0.97	0.79, 1.20	0.795
Likelihood ratio test					0.1610

Reflexivity Statement

Study conceptualisation

1. How does this study address local research and policy priorities?

The trial's research questions and outcomes were chosen in consultation with Mali's Ministry of Health and Social Affairs to answer questions of key concern as they develop and implement national health policies. How to optimise community health worker service delivery in rural Mali to reduce child death and improve access to essential services is a key local policy question. We assessed heterogeneous treatment effects by potential modifiers that are relevant to the country's community health strategy, including cluster population size, distance to health center, and household wealth. We also embedded a costing analysis into the trial because whether the intervention would be affordable at scale was a key concern of the government.

2. How were local researchers involved in study design?

Malian authors KK, DD, and MG were involved in study design.

DD (LMIC researcher) and AJ (HIC researcher) are founding members of Muso, and initially designed and tested the proactive CHW home visit intervention in Bamako, Mali. Following promising observational results in this peri-urban context, DD and AJ and the Ministry of Health and Social Affairs sought to implement and test the intervention next in a rural Malian context and, specifically, isolate and evaluate the effects of CHW home visits from the other health system enabling co-interventions (professional CHWs, upgraded primary care clinics, user fee removal).

MG is the chief doctor at the Bankass referral hospital. He selected and prepared the study sites in collaboration with Muso's Amadou Beydi Cissé (Malian researcher) who is an author on the trial's primary under-five mortality outcome paper (submitted) and is acknowledged in this paper.

KK was brought on board to pursue the trial as one of three co-PIs and the founding director the Muso's research department. He was assisted by Dr Belco Poudiougou (Malian researcher) who passed away in 2017 and is recognised posthumously in the acknowledgements.

Research management

3. How has funding been used to support the local research team(s)?

Muso received unrestricted and restricted funding to support this trial, which paid the salaries or consultant fees of both LMIC and HIC research team members. Fee support levels were set to comply with the salary scales of the academic researcher's institution, prorated to the time contributed to the trial. We acknowledge the considerable discrepancy between the salaries and/or consultancy fees benchmarked in LMICs versus HICs.

Data acquisition and analysis

4. How are research staff who conducted data collection acknowledged?

NK and MBT (Malian researchers) are acknowledged in third and fifth authorship positions, respectively. They supervised data collection teams (of 120 surveyors total) at every annual survey round, from baseline, beginning in December 2016, to year three, ending in April 2020. Other Malian research staff who supervised data collection teams or coordinated field activities during a portion of the trial period, such as Belco Poudiougou, Yacouba Samaké, Mahamadou Sogoba, and Saibou Doumbia, are recognised in the acknowledgements of this paper and as authors on other trial papers (submitted or drafting) that are aligned with their interests and contributions to the trial.

5. How have members of the research partnership been provided with access to study data?

All research partners who were involved in data quality assurance/quality control, cleaning, management, preparation, and analysis had access to study data.

During the trial period, Muso built a data warehouse. Now that survey data sets have been fully cleaned and locked, we are uploading them to the data warehouse so that all research partners with authorised access can more readily access and extract data for their planned and exploratory analyses.

6. How were data used to develop analytical skills within the partnership?

For this secondary trial endpoint paper, an early career researcher CW developed her analytical skills in writing the statistical plan and executing the analysis under the guidance of her more senior statistical advisor, CL (HIC researcher). CW is a HIC researcher (from a HIC and affiliated with a HIC university as a PhD candidate) and has been based in Mali since 2015 when the trial was designed. For the primary under-five mortality outcome, a HIC post-doctoral early career researcher (ET) was given the lead in planning and executing the analysis under the mentorship of her more senior HIC advisor (JL).

We acknowledge that precedence has been given to HIC researchers over LMIC researchers when it comes to conducting the analyses of prespecified primary and priority secondary trial outcomes, such as this one. When the consortium was first established, HIC (affiliated and/or based) researchers were recruited as trial statisticians, due (at least in part) to the systemic privilege that grants them preferential access to resources, such as training, mentorship, and time, required to analyse trial data.

However, there are currently 20 trial analyses/papers planned by the consortium, including other secondary endpoints, nested/sub-studies, and exploratory or secondary data analyses. These are in various phases of development, from conception to data preparation, analysis, drafting, or

submitting. Early career Malian researchers, including NK and MBT, are leading analyses and/or contributing important analytical portions to these works in progress.

Furthermore, Malian researchers led data quality assurance/quality control throughout the trial, developing their skills in this area. Malian data managers at different points during the trial, such as Idrissa Kamara, Lamine Guindo, Mahamadou Sylla, are recognised in the acknowledgements of this paper and as authors on other trial papers (submitted or drafting) that are aligned with their interests and contributions to the trial.

Data interpretation

7. How have research partners collaborated in interpreting study data?

We convened workshops with various stakeholder groups, to present and discuss/interpret results, virtually or in person, in French. The trial primary and secondary outcome results, including those reported in this paper, were presented and discussed in group with: the Muso leadership team (virtual); the Muso research department (virtual); the entire Muso team (virtual); district level health and political authorities (in person); and community representatives separately per health catchment area (in person). Community representatives included village chiefs and their advisors, women's and youth association leaders, religious leaders, local political and health authorities, study CHWs, and dedicated CHW supervisors. With Ministry of Health and Social Affairs research partners and authors (MC, MB, MG), we held one-on-one in person meetings to discuss and interpret results. For this secondary trial endpoint analysis/paper, the lead analyst/author (CW) held an additional authors contribution focus group with DD, NK, and MBT to discuss and interpret results in depth.

All drafts were circulated to all Malian authors in French, using a free online translation tool. Their input, in French, was solicited verbally, through comments/annotations, or via direct writing, on specific questions specially articulated/highlighted given their expertise, as well as more generally.

Drafting and revising for intellectual content

8. How were research partners supported to develop writing skills?

For this secondary trial endpoint paper, we used translation, virtual, verbal, and written methods to contribute to writing, as described in #7. Malian authors were invited to write sub-sections, with a focus on the methods and discussion.

Similar to what is described in #6, this is one paper of many related to this trial. Malian researchers are leading and will lead the writing on other forthcoming papers. For example, NK is leading a complementary paper examining changes in reported barriers to care for sick children across arms compared to baseline.

9. How will research products be shared to address local needs?

In collaboration with Muso's technical assistance department and our ministry partners, we will work to facilitate the process of translating trial findings to action and policy recommendations for Mali's community health plan. For one, we will write and share policy memos (in French and English) to accompany the scientific publications.

Our Mali dissemination plan, developed with our Ministry of Health and Social Affairs authors (MC, MB), began with the local and district level dissemination workshops described in #7. Next, we will support the Bankass district health and political authorities to present the results at regional level workshops, with the Regional Health Directorate and its partners in health planning and implementation in Mopti. Next, the regional health and political authorities will present the results at national level workshops, with governmental and non-governmental stakeholders and coalitions making decisions about community health in Mali. These workshops will enable local policy leaders to integrate the study findings to guide the implementation of Mali's national community health strategy and health system strengthening efforts.

We have started the process of identifying francophone conferences/symposia in Mali and the West African region but need to renew our efforts to select and submit trial results to these fora. We will also consider which trial papers to submit for publication in African regional journals.

Authorship

10. How is the leadership, contribution and ownership of this work by LMIC researchers recognised within the authorship?

Half (7 out of 14) of the authors on this paper are Malian (LMIC) researchers, and the other half are HIC researchers. The second and third authors are Malian (LMIC) researchers.

KK (a male, senior, LMIC researcher, based in Mali) and AJ (a male, senior, HIC researcher, based in USA) are trial co-PIs. On the trial's primary under-five mortality outcome paper (submitted), they are co-senior authors to reflect this. However, in KK's West African francophone academic environment, it is preferential to be among the first three authors rather than the senior, co-senior, or penultimate author. Thus, KK is second author on this paper, after CW (a female, early career, HIC researcher, based in Mali) in the first position who led this analysis and writing.

KK is followed by NK (a female, early career, Malian researcher) in the third authorship position, to recognise her invaluable contributions to data acquisition, including supervising data collection and participating in data quality assurance/quality control, cleaning, and management.

Three Ministry of Health and Social Affairs partners (MC, MB, MG) are recognised as middle authors to recognise their role as co-owners of this research and their involvement in intervention oversight and the interpretation of results.

11. How have early career researchers across the partnership been included within the authorship team?

Early career researchers have been given prominently places in the author list, including the first (CW), third (NK), and co-senior positions (ET).

An early career, female, HIC researcher was given the lead on this secondary trial endpoint analysis and is the first author (CW). She was based in Mali throughout the trial, with periods in the UK where she is affiliated with LSHTM as a PhD student. Her PhD advisory committee members are included among the authors on this particular trial paper (KK, AJ, BG, DC, CL). Other early career researchers are included among the authors, including two Malian researchers (NK, MBT).

12. How has gender balance been addressed within the authorship?

Five out of the 14 authors are female (CW, ET, NK, JL, CL), including the first, co-senior, and penultimate senior authors who led the analytical work for this paper. We recognise that only one female author is Malian (NK).

Training

13. How has the project contributed to training of LMIC researchers?

This is one of the world's largest trials in community health. Being a partner in this research provided great professional development for LMIC and HIC researchers alike.

The trial provided on-the-job training of Malian researchers in survey development, good clinical practice and research ethics, data collection and quality assurance/quality control, and data cleaning and management. We acknowledge that we are in the early stages of leveraging the trial to contribute more to the training of Malian researchers in data analysis and paper writing. This includes research question generation and protocol writing, for secondary analyses of trial data. Furthermore, trial collaborators who participated in intervention implementation, such as CHWs and programme managers, supervisors, or administrators could have participated more fully in the research process had the project contributed more to their training in the fundamentals of research methods and data.

Muso has provided funding for LMIC (and HIC) researchers to pursue professional development opportunities such as international conferences attendance and higher education degrees, including in statistical analysis.

Infrastructure

14. How has the project contributed to improvements in local infrastructure?

Since the trial was designed, Muso's research department has grown to what is today 13 West and Central African research investigators and evaluators.

Muso has continued and will continue to support the Ministry of Health and Social Affairs in implementing the co-interventions across both arms, including 8 refurbished and reinforced

primary care clinics (including one pilot area), 168 professionalised CHWs and their 10 dedicated supervisors, and user fee removal, beyond the end of the trial.

Governance

15. What safeguarding procedures were used to protect local study participants and researchers?

Within each catchment area, we held public consultation meetings with community representatives, including village chiefs and their advisors, women's and youth association leaders, religious leaders, and politico-administrative authorities (such as mayors, PHC directors, and Community Health Associations), where we discussed and obtained verbal permission to conduct the trial.

When violent conflict arrived in the study area, a detailed security plan established protections, contingencies, and continuity of services to protect study participants, providers, and researchers from the dangers of conflict. We also adapted health-care delivery and research survey procedures to protect health and safety throughout the Covid-19 pandemic.

The ProCCM Trial engaged three different oversight bodies to protect study participants: the local ethics committee in Bamako, an independent clinical research associate from Senegal, and a data safety and monitoring board, which included a senior Malian researcher. All survey respondents provided written, informed consent or assent at their first enrolment. Any individual, regardless of residency, survey enrolment, or arm assignment, who sought care from study providers was eligible to receive health-care throughout the trial.

We held fortnightly consortium meetings throughout the trial period. These were conducted primarily in English, which is the first language of the HIC researchers and the third language of the Malian researchers. As a result, HIC (based/affiliated) researchers in the partnership tended to be overrepresented in the consortium meetings and likely more comfortable speaking. We acknowledge that there is improvement to be made in the equity, diversity, and inclusion of participation at research consortium meetings.
